# Informing the Cannabis Conjecture: From Life’s Beginnings to Mitochondria, Membranes and the Electrome—A Review

**DOI:** 10.3390/ijms241713070

**Published:** 2023-08-22

**Authors:** Alistair V. W. Nunn, Geoffrey W. Guy, Jimmy D. Bell

**Affiliations:** 1 Research Centre for Optimal Health, Department of Life Sciences, University of Westminster, London W1W 6UW, UK; gwg@theguyfoundation.org (G.W.G.); j.bell@westminster.ac.uk (J.D.B.); 2 The Guy Foundation, Beaminster DT8 3HY, UK

**Keywords:** phytocannabinoids, mitochondria, membranes, bioelectricity, thermodynamics, quantum mechanics, epilepsy, cancer, cannabidiol

## Abstract

Before the late 1980s, ideas around how the lipophilic phytocannabinoids might be working involved membranes and bioenergetics as these disciplines were “in vogue”. However, as interest in genetics and pharmacology grew, interest in mitochondria (and membranes) waned. The discovery of the cognate receptor for tetrahydrocannabinol (THC) led to the classification of the endocannabinoid system (ECS) and the conjecture that phytocannabinoids might be “working” through this system. However, the how and the “why” they might be beneficial, especially for compounds like CBD, remains unclear. Given the centrality of membranes and mitochondria in complex organisms, and their evolutionary heritage from the beginnings of life, revisiting phytocannabinoid action in this light could be enlightening. For example, life can be described as a self-organising and replicating far from equilibrium dissipating system, which is defined by the movement of charge across a membrane. Hence the building evidence, at least in animals, that THC and CBD modulate mitochondrial function could be highly informative. In this paper, we offer a unique perspective to the question, why and how do compounds like CBD potentially work as medicines in so many different conditions? The answer, we suggest, is that they can modulate membrane fluidity in a number of ways and thus dissipation and engender homeostasis, particularly under stress. To understand this, we need to embrace origins of life theories, the role of mitochondria in plants and explanations of disease and ageing from an adaptive thermodynamic perspective, as well as quantum mechanics.

## 1. Introduction

“Conjecture: A conclusion that is based on information that is not certain or complete (Collins)”.

For many years, there have been three primary questions: can phytocannabinoids work as medicines, are they safe and how do they work? The first two, perhaps, apart from 1000s of years of observational data [1], have now been answered to some degree for botanically derived cannabidiol (CBD) as a treatment for epilepsy [2]. The third, however, especially for non-psychoactive compounds, such as CBD, has not—despite the discovery of the so-called “endocannabinoid system” (ECS); CBD only interacts weakly with it and binds to many different components in the cell, making a precise mode of action difficult to pin down [3]. In fact, the plant generates many different compounds, of which tetrahydrocannabinol (THC) and CBD are the best described, and it is likely that the mix of compounds, via the so-called “entourage” effect, could be key in their efficacy [4]. However, certainly from a one target one receptor pharmacological point of view, this tends to make refining a mode of action even more difficult, but there are generalisations that as a group, they tend to be anti-inflammatory and have anti-pathogen and anti-cancer activity. This does indicate that as far as the plant is concerned, they are a part of its stress resistance arsenal [1]. Understanding this could be key. There are also three far deeper questions; why would plant compounds even work as medicines in animals at all, what is it about disease that they might be able to correct, and thirdly, what is it about their structure that gives them this capacity?

The first is hinted at because mitochondria are central to energy dissipation in plants, and thus are key in the stress response and thus management of reactive oxygen species (ROS) [5]. It would be surprising that the evolution of any stress adaptation compounds would not modulate these organelles.

The second is to embrace the underlying evolutionary thermodynamic principles that may have led to life and follow it through to what it implies about ageing and disease today—and thus medicine. It is generally agreed that life is a replicating self-organising “dissipative” structure that exists far from equilibrium, in effect, it falls out of the field of adaptive thermodynamics and entropy and the inevitable propensity for energy to equilibrate and disorder to increase. As was said many years ago, life is just a small-organised “negentropic” lump of matter that obeys entropy by “dissipating” energy potentials—a concept promoted by Erwin Schrödinger [6]. Equally, Albert Szent-Györgyi famously said that “life is nothing but an electron looking for a place”, which epitomises the role of redox in biology; he also was pivotal in unlocking, even if he was not entirely correct, the workings of the Kreb’s cycle [7]. Once the dissipative explanation is accepted, this then leads to a further insight about what else the dissipation could be doing; dissipation is largely maintained by the movement of ions, which creates charge separation and thus electric fields—putting electrophysics and membranes centre stage in biology. The importance of fields and bioelectricity has long been suggested, but not always accepted, but evidence continues to build of its centrality in life [8]. It could therefore be said that life can be defined by its ability to adapt, but critically, it must exist in a sweet spot where modulation of dissipation is key, which must involve control of redox and electric fields, and critically, membranes. Life has evolved highly complex systems to maintain its membranes—in particular, their fluidity in a very narrow range—and can affect the diffusion of molecules such as oxygen. Critically, oxidative stress damages membranes and reduces their fluidity, implying a strong relationship between the two [9].

The third is hinted at from the angle that many plant compounds do appear to have evolved as sunscreens and antioxidants, as well as interacting with mitochondria and showing evidence that they can uncouple. In effect, they could be part of a mechanism to manipulate the dissipation of energy to restore homeostasis. It could therefore be argued that this core ability has, through millions of years of evolution, become part of a general stress response system [10]. Equally, evidence is also building that photosynthetic organisms have evolved not only highly efficient photonic harvesting systems that are based on quantum principles such as vibronic coupling, but also may use something similar in photoprotection to divert energy away to protect light harvesting systems. These systems need to react very fast and include redox-active cysteine residues to fine tune resonance coupling between excitons and pigment vibration to divert excess energy towards a quenching site, which is known as redox-dependent exciton steering [11]. This of course raises not only some interesting questions about the extent that plant secondary metabolites interact with the plant’s mitochondrial stress resistance system, but also whether some deeper quantum principles may be in play in how they might work.

What is certainly clear is the central role of the mitochondrion in just about all aspects of modern eukaryotic life, ranging from inflammation, management of oxidative stress, sex, to ageing and disease, as well as death, and thus the role of electron flow and the proton gradient. This suggests that life may have started in something like an alkaline thermal vent full of crystalline cells driven by the energy differential from hydrogen flowing up from the underlying substrata and interacting with carbon dioxide in sea water [12,13].

Given the centrality of mitochondria in modern multicellular life, it would be extraordinary if phytocannabinoids did not modulate mitochondrial function. Unfortunately, it seems that research investment into the effects of secondary plant metabolites on mitochondria in plants has only ever been at a fraction of that in animal cells, but it is certainly the case that many of these compounds do modulate mitochondrial function in animal cells [14]. The “why” thus derives from the commonality of life to balance bioenergetics with stress adaptation in relation to the movement of electrons and energy, and the “how” derives from the manipulation of a sweet spot in homeostasis that certainly in complex eukaryotes will involve membranes, especially of those in the mitochondrion.

However, this is only part of the problem. To truly understand how these compounds may work also requires a deeper insight into what “disease” is and why it occurs, which could also fall out of a deeper understanding of the origins of life. For example, the concept of “inflammaging” embraces the idea that as more complex organisms age, they lose their ability to manage the inflammatory response as their ability to adapt via hormesis decreases, but hormesis can, to some degree, modulate this process. There is in effect a hormetic “goldilocks zone”, but with age, it narrows. Much like the flight envelope of an aircraft, there is a metabolic envelope for life, but as we age, it narrows. And to borrow another term from aviation, it eventually narrows so much that we eventually hit “coffin corner”. It may therefore be possible from the thermodynamic dissipative perspective to gain insight into what inflammation and hormesis actually represent [15]. It is thus significant that simple approaches, such as diet and exercise, can have profound effects on most common diseases, with mitochondria playing a significant role [16]; these have the ability to widen our metabolic flight envelope.

Overall, this implies that lipid-soluble redox-capable compounds could be affecting signalling and bioenergetics via a very basic mechanism that restores homeostasis and optimal dissipation in a dysfunctional system. This could provide a simple explanation of how they work in both the plant and an animal and could well tie in with the re-emergence of interest in bioelectricity. At a deeper level, if simple modulation of, say, membrane structure could alter the “quantum underground”, this could be something to explain a more generalised protection mechanism, for instance, involving vibrational coupling to diffuse the energy in over-excited systems towards a safer quenching operation. Any shift in membrane structure will induce a conformational shift in just about every protein in a cell, which, it has to be remembered, are constantly oscillating due to thermal energy. This could involve ion channels, membrane/cytoskeleton adhesion points, and large complexes like those found in the mitochondrion. If, as many suspect, electron as well as proton transport are reliant on quantum tunnelling, then the smallest shifts in vibrational coupling relating to distance will have profound effects. As indicated, in plants, redox-dependent exciton steering could well be important in photoprotection [11], but photons are not the only source of potentially excited electrons; chemiexcitation also occurs in animals too through metabolism [17]. It is also becoming clear that the photoprotection system in plants is also reliant on maintaining membrane fluidity by minimising oxidative loss of fluidity [18], which is also likely to be true in animals. Evidence is that diets high in plant-derived antioxidants, both water- and lipid-soluble, can maintain membrane fluidity and inhibit the production of oxidised lipids, especially from polyunsaturated fatty acids that are key in inflammation [19].

It could be argued that life has evolved around the core properties of molecules like CBD to dissipate excited states, which would imply, de facto, they must also modulate bioenergetics and membrane fluidity. Inflammation can be viewed from the chemiexcitation viewpoint as a mis-flow of electrons disrupting homeostasis that induces a feedback mechanism, which if it does not correct leads to what might be described as “disease”. It could therefore be said that we age and die because our systems slowly lose the ability to adapt, and in this respect, health is about the ability to maintain this adaptability. To some degree, the right amount of stress, embraced by the concept of hormesis, does seem to stimulate this adaptability, as without it, we age faster [20]. This might suggest that compounds like CBD can not only act to “deexcite” a system but can also stimulate it to enhance adaptability by restoring a homeostatic feedback mechanism. From the thermodynamic point of view, this could be related to the restoration of membrane fluidity to its “sweet spot” and optimal dissipation. In a multicellular organism, this could enhance cellular cooperativity, but it could also explain how it could be anti-pathogenic by disrupting membranes of single-celled organisms. This implies that due to concentration effects, it is likely that biological effects could be at least biphasic, with a low dose doing something different to a high dose.

This is of course also highlighted by the well-described concentration effects of both THC and CBD, in particular of the former, when used in higher doses, and their relationship with known molecular targets, such as channels and receptors—and thus a conventional pharmacological approach. Critically, they certainly appear to modulate many potentially clinically relevant receptors and channels at lower doses as well (reviewed in [21]). Thus, part of their mode of action can be explained by the conventional pharmacological approach. However, as far as animals are concerned, they are xenobiotics, and the pathways involved in the xenobiotic response are involved in hormesis [22]. So, the key here is that as it was likely that in terms of evolution, membranes came first, followed by the evolution of complex ion channels and receptors that helped to control ion flow and membrane potential, which likely meant that the earliest proteins evolved around a basic principle that already existed, for instance, that membranes already had some permeability to ions. It is thus probable that the channels and the surrounding membrane composition co-evolved—probably from simple peptides [23]. Controlling calcium, in particular, may have been pivotal, which explains why our resident intracellular descendent of a prokaryote, the mitochondria, is key in extant calcium signalling [24]. In fact, it has been suggested that the origins of calcium-triggered membrane depolarization in modern cells evolved as an emergency response due to membrane damage [25]. In short, when considering how lipophilic compounds work, both their direct effects on membranes and associated proteins ought to be taken into account, because it is likely that life has evolved sensitive sensor-homeostatic systems to detect the presence of potentially disruptive agents and transmit stress signals. This could of course provide some interesting insight into the evolution of the endocannabinoid system in animals, as well as the plant cannabinoid system, and why compounds from the plant work as medicines.

This paper is split into five sections, the first provides some background insight into origins of life and the mitochondrion and how this leads to an understanding of ageing and disease, while the second reviews the evidence linking phytocannabinoids and mitochondria. The third section focusses on the link between phytocannabinoids and calcium signalling; calcium is an easily measured ion that likely reflects the dissipative aspects of cellular function, as it plays a dual role in signalling and energy manipulation. In the fourth section, we look at some different ways of viewing how they might work in disease. In the final section, we outline a big picture approach to explain how they might work and apply it to some key diseases, including a suggestion about their role in how life adapts to stress in relation to bioelectricity.

## 2. From Thermal Vents to Ageing and Disease

In this section, we provide a brief overview of the history of life and current theories that seem to explain why it is the way it is. This is key in helping to understand why it ages and eventually fails and how plant compounds, such as the phytocannabinoids, might work. For example, the “hard” questions in biology are still how it started, what is consciousness, what is ageing and what, for instance, is cancer. Tellingly, which could be related to answering these hard questions, we are still struggling with some of the basics, for instance, is biology using significant quantum effects [26], and what is the role of electromagnetic fields [8]? Furthermore, the latter concept is tied closely to bioelectricity and how life programs its shape; for example, genetics may play less of a role than thought, and it could be more to do with the morphogenetic field [27].

How we think about problems is important, and embracing disciplines outside of mainstream medicine maybe informative, for instance, what can the origins of life theory tell us about medicine today? The best explanation is that life is a self-organising replicating far from equilibrium dissipative system, as this follows the fundamental laws of quantum mechanics and thermodynamics. Many of these ideas were developed by Ilya Prigogine from the second law and dissipative processes in quantum systems [28]. Today, the concept is now well established [29,30]—and has been tested in model systems, indicating that self-replication can emerge in non-biotic systems, which display dissipative adaptation [31]. In short, the underlying maths does seem to support how a complex non-life system, in an energy gradient, can become organised around dissipation and become life. Given the importance of fields, it is even possible that the movement of charge, say, in a thermal vent, which would have generated fields, was also pivotal in organising life—hinting at how the morphogenetic field code could have arisen [32].

### 2.1. Thermal Vents to Mitochondria

As the physical principles explaining what life could be in terms of the laws of quantum mechanics and thermodynamics are now becoming more accepted, this raises the question, does this help in identifying where it might have started? One essential ingredient seems to be a large energy potential. With this in mind, we can say that extant life evolved from its ancestors and thus, although it is apparently complex today, this complexity is built on something much simpler, which for billions of years was prokaryotic. So, it is likely that the core metabolism and structure of modern life will reflect its beginnings, which could well be this energy gradient. For example, it is possible that one of the earliest catalytic systems involved a very simple nickel-iron hydrogenase, indeed, it is possible to design one with 13 amino acids, which can extract energy from a hydrogen gradient—today of course, these proteins are vastly more complex [33]. The key in this is that life will have evolved around the core properties of the earliest existing pre-biotic molecules. Strip away the complexities of proteins, and we are left with aromatics as cofactors, and core metal-based compounds, all of which have some key quantum properties that enable the transfer of energy via electrons and protons. Critically, most are imbedded in membranes.

This energy gradient is found in all life today and relies on a flow of electrons and the generation of ion gradients across membranes, with the largest being mainly comprised of a proton gradient across the inner membrane of the mitochondrion, which is of course the basis of the chemiosmotic theory [34]. One of the strongest contenders for a starting place for life, which today exhibits the appropriate non-equilibrium geochemistry, are alkaline thermal vents, where a flow of hydrogen from the Earth’s crust can meet sea water containing carbon dioxide. Although there are other theories, which we will not cover here, the alkaline thermal vent theory does fit well with the existence of the Kreb’s cycle, chemiosmosis, as well as the generation of membranes. Critically, by simply reversing the direction of the Kreb’s cycle to a biosynthetic mode, one can perhaps see an echo of how life started—dissipation of energy is achieved by forming more complex structures. At some point, life managed to reverse the direction by using other energy sources to generate its own gradient. Later, modern eukaryotic cells arose by some form of symbiosis between an Archaean and a bacterium, with the latter becoming the mitochondrion [7,12,35]. Critical in the transition from metabolism driven by the thermal vent to something that was independent was an evolution of membrane structure that became less leaky. It is likely that early life was not very efficient at all, it did not need to be, but with time, it became more sophisticated as the original energy source became more restricted [36]. The structure of membranes is thus critical to all life and underlies the process of chemiosmosis.

In terms of timings, it is thought that the progenitor of the mitochondrion, an α-proteobacterium, underwent endosymbiosis around 1.5 Gyrs ago to give the eukaryote, and later, a symbiotic event with a cyanobacterium gave rise to the progenitor of the chloroplast, around 1.2 Gyrs ago—giving plants three genomes [37]. Interestingly, the latest research might indicate that an “Asgard” Archaean, which has both a form of a cytoskeleton and can exude tentacle-like protuberances, could have been one of the key candidates as it may well have already had more complex membrane structures [38]. The mitochondria of plants are thus very similar to those in animals but do have some key differences, with some extra subunits in components of the electron transport chain (ETC), giving oxidative phosphorylation a few extra functions as the whole system has to be integrated with chloroplast activity [39].

It is thought that photosynthesis itself probably evolved after life began, quite possibly due to the presence of long-wavelength light generated by an alkaline thermal vent, but still using the basic evolved chemistry; the ETCs used in photosynthesis and respiration are very similar, and it is only the source of the energy that is different [40]. Given the importance of quantum mechanics in biology, in particular electrodynamics, the hot thermal vent theory also lends itself to other factors, such as electromagnetic fields and photons being important. In a sense, life could have begun with some kind of morphogenetic field [32]. Certainly, the role of fields in life is gaining much more attention [8], and it is pretty well accepted that life is electrical [41,42]. In short, when considering homeostasis, we also must consider the role of fields, as life is all about dissipating energy by moving charge.

The importance of charge is clearly seen in the very high electric field generated by the high voltage across membranes, for instance, it approaches 10 million volts per metre, which in turn generates a force on the membrane—which likely contributes to electromotility [43]. Thus, biomembranes can be viewed as thin capacitors, and external charges can influence the state of the membrane via a process of “electrostriction” and can alter charge flow [44]. This might help explain the well-observed effects of electric and magnetic fields on biology [45].

The bottom line is that thermal vents provided all the conditions for some kind of self-replicating dissipating system to evolve, which was based on the cycling of ions through a membrane, creating both static and dynamic electromagnetic fields. As plants and animals are eukaryotes, with common ancestry, they still share the basic dissipating systems, including the mitochondrion—where movement of electrons and protons is central—and very likely bioelectric fields (which we discuss later). Critically, anything that alters the membrane structure will thus alter its electrical properties, as well as the dissipating self-organising process that defines life. This further strengthens the case that life evolved because of and is harnessing and potentially amplifying some aspects of quantum mechanics not always thought possible in the “warm and wet” milieu of life, which is tightly linked to adaptive thermodynamics.

### 2.2. Homeostasis or Die—What a Dissipative Theory Might Tell Us about Ageing 

What is clear is that most individual organisms, whether they be plant, fungi, prokaryote or animal, die. If they are not killed, they still age and stop working. For most, this is due to one system or another losing its integrity, and is usually due to its components becoming damaged, and not being replaced to the point that homeostasis fails. So, from a simple perspective, the ability to prevent damage, detect it and repair it dictates lifespan. When a system starts to malfunction, we call it disease. Indeed, it seems that programmed cell death (PCD), or “dying for the greater good”, may have evolved very early in evolution; prokaryotes do it—as they are often part of a multicellular colony [46].

Key in death, for both prokaryotes and eukaryotes, is the loss of the membrane potential [47]. Equally, prokaryotic membrane potential is key in just about every aspect of prokaryotic life, ranging from resistance to antibiotics to ion-channel-based bioelectrical signalling in biofilms, the latter having many similarities to that seen in eukaryotes [48,49]. Of course, turning this around simply implies that bioelectrical signalling evolved in prokaryotes, and has become more complex in eukaryotes. This is the same for the existence of PCD, which seems almost counterintuitive for single-celled entities. However, more recently, the existence of PCD has reignited the discussion around whether ageing is adaptive or not; is death just due to outside influences and/or a failing system, or is it the result of something far more fundamental? This is important, as it might shed light on why disease occurs, and what compounds like CBD evolved for.

To help answer this question, one explanation may come from the dissipative explanation of life and the maths underlying it. For example, if life on the planet is viewed in its entirety, the concepts of hormesis (stress-induced adaption and a biphasic dose effect) and inflammation (the process whereby stress-induced damage is repaired) could be interpreted as flipsides of the same process and to some degree, scale-invariant as long as life as a dissipative process at the global scale continues. Implicit in the concept is that natural selection will remove failing systems to ensure functional ones survive and that information will be retained to enable this to happen; adaptive thermodynamics indicates this process could have become “hard-wired” in biology. This would suggest that proteins, organelles, cells, organisms and entire species are dispensable and removed if they fail to maintain dissipation and potentially damage other functional systems. In effect, we can apply inflammation and hormesis in relation to the Gaia hypothesis, where all life is interconnected at the global level. Inflammation can thus be described as a repair process whereby old damaged structures are removed and, using prior information, rebuilt; if the stress is too much and they cannot adapt, in effect, they are not robust enough, they fail [15]. This of course should be a worry for humans; we are dispensable at the global scale.

In effect, biology emerges from self-organisation of existing compounds and chemistry, creating order from disorder; it is important to view the interaction of its networks both from a bottom-up and from a top-down aspect—which requires a systems biology approach [50]. The discovery of reflexively autocatalytic food-generated networks embedded with prokaryote metabolism indicates an emergence of thermodynamically driven autocatalytic chemical networks before proteins and RNA, hinting at the transition between earth chemistry and life [51]. Thus, for an individual structure to survive, it must dissipate; modern organisms are simply more complex versions of this initial physics-driven system at the beginning of life and subject to the same laws.

### 2.3. Optimal Health and Disease

The above discussion hints that life operates in a Goldilocks zone, which is embraced by the concept of “hormesis” and the ability to adapt to change. If stress induces damage, too much will kill, but a smaller amount will induce adaptation. In effect, what does not kill you makes you stronger, and we would add, longer lived and smarter. Why? If we accept the idea of adaptive thermodynamics and dissipation coupled to natural selection, stress will induce natural selection of the most stable systems and the removal of the less fit, and if information is encoded that enables new structures to be built, then a system will adapt. At the Earth global level, this is clearly seen as those species most adapted to whatever a new set of circumstances is thrown at them will survive. In a way, life will explore the “phase space” of possibilities. The same is true of an individual but within a narrower band, and operates at the cellular, sub-cellular and molecular level. A classic example of this is mitohormesis, where sublethal stress results in increased ROS but induces both the turnover and upregulation of a healthier population of mitochondria—and underlies the many benefits of exercise, calorie restriction and a diet high in dietary phytonutrients [52].

Originally the term “dis-ease” simply meant lack of ease or comfort but has now come to mean a disorder of structure or function of all or part of an organism. This can be induced by environmental factors and pathogens but also internal errors. Ageing is associated with a progressive degeneration of systems, with declining robustness and, to some extent, reducing biological fitness and increasing frailty. So not only does ageing on its own increase the likelihood of definable disease, but it makes it more likely that external factors can induce it. However, there is still some disparity between researchers on what it is, including whether or not ageing is a disease in itself, and whether we need to shift from a disease-orientated to health-orientated model and whether or not, due to the interconnectedness of body systems, we need to take a holistic approach [53]. There is thus also a need to define what we mean by living in “optimal health”. Clues for this may come from the observation that optimal human functioning does require physical activity across the lifespan, the “use it or lose” paradigm [54]. In short, most life requires hormesis, which suggests a definition: 

“Optimal health occurs when an organism is able to easily adapt to those stresses it evolved in, but this adaptability is only maintained by regular exposure to these stresses within an adaptive zone, which narrows with age due to a gradual loss of information due to entropy and no purifying natural selection of structural components”.

If life is effectively a negatively entropic structure that is dissipating energy to bring about equilibrium, then for this to happen, the structure must be assembled correctly and stay functional, and any damage could hinder this; through time, via natural selection and evolution, these structures have become ever more complex by incorporating heritable information. Much of this information could be held in bioelectric fields, indicating that we have to rethink the role of genetics in, say, development—as indicated by the importance, right across kingdoms, of the v-ATPase [55]. It can be argued that as the evolution of intelligence is a natural consequence of life trying to “outwit” a challenging environment, there is a downside; taken to its logical extreme, life can remove the very factors that drove its evolution to complexity. This is of course inherent in the concept of hormesis. Thus, somewhat paradoxically, many humans are now reducing both their lifespan and healthspan, resulting in morbidity expansion. This appears to be caused by a poor lifestyle due, in part, to the removal of a key component in maintaining optimal health: mild stress [56].

It could therefore be said that “optimal health” is achieved when an organism exists in the middle of its hormetic Goldilocks zone, too little stress reduces its robustness, while too much will kill it. Disease can be said to occur at the extremes, and, as the organism ages, this “metabolic flight envelope” zone narrows as its ability to adapt and maintain dissipation decreases. Eventually it narrows so much that homeostasis cannot be maintained, and it dies.

### 2.4. The Nuts and Bolts of Ageing and Death

Ageing and disease are thus fundamental to maintaining the dissipative process of life at the global scale. It is informative to view the evolved mechanisms that underly it from unicellular to multicellular organisms. For prokaryotes, this seems to be related to a failure of proteostasis and the ability to clear misfolded proteins. For more complex species, histone/chromatin stability became important. Then, in early eukaryotes, it seems that the loss of nuclear stability and proteolytic systems, ROS damage, mitochondrial failure and nutrient sensing failure became key. Finally, in multicellular life, loss of intracellular communication, accumulation of damaged cells, loss of regenerative powers and DNA damage became key. In effect, this is a multi-layered system, starting with the accumulation of misfolded proteins as the most ancient, then epigenetic changes, then mitochondrial dysfunction associated with ROS and on up to a loss of multicellular cooperation and senescence [57].

At the multicellular level, perhaps the best example of this is embodied by the mitochondrial lysosomal axis theory of ageing, which simply stipulates that as mitochondrial function degrades, this reduces the ability of the lysosomal system to remove damaged mitochondria, in effect, leading to a vicious cycle with rising levels of oxidative stress; fundamentally, this is down to the ability of mitochondria to produce ATP [58]. It could also be argued that as mitochondria can act as net sinks of ROS [59,60,61], their declining anti-oxidant capacity could further add to a vicious cycle

It has long been thought that maintaining mitochondrial function is a key component of healthy ageing, as it is intimately linked to inflammation [62]. In support of this, new data, gained from studying patients with primary mitochondrial defects and models where mitochondrial function is inhibited, seem to indicate that if mitochondrial function is minimally reduced, the cell resets its metabolism by shifting more towards glycolysis, resulting in a hypermetabolic state. In effect, this increases its energy production and becomes less efficient—but this comes at a cost of increased stress and epigenetic changes, accelerating the ageing process. Patients with primary mitochondrial defects usually have reduced lifespans, are easily fatigued by exercise and have a resting VO_2_, on average, 30% higher than healthy controls [63]. This is of course almost the exact opposite of what happens in calorie restriction.

### 2.5. Lifespan Determinates

So, what this suggests is that reproduction “resets” a nascent organism’s structure, via a process of natural selection, to something that is optimally functional. However, with time, this structure slowly degrades and its lifespan, notwithstanding predation, accidents or major environmental challenges, is determined by how well it can maintain this structure. The survival of the species is thus a balance between individual survival and that of its offspring and the ability to evolve if circumstances change. This prompted the concept of the disposable soma theory of ageing, where lifespan is determined by a balance between maintaining the soma and reproduction, and of course, what the environment does and predation. The antagonistic pleiotropic theory of ageing, in slight contrast, suggested that genes that aided in survival when young may be detrimental when an organism ages. A further complicating factor, which is often overlooked, is that pathogens themselves may modulate lifespan for their own benefit [64]. In a sense, ageing, and thus disease, is a balance of natural selection and repair, modulated by environmental factors.

Whatever the precise theory, across life, there is a large range of lifespans, with each species adapted to its niche—some species having short lives with high reproduction rates, while others, as individuals, live a long time, but produce a smaller number of offspring, although there are exceptions in both cases. Generally speaking, evolved traits that can reduce environmental/predation effects, such as large size, intelligence or the ability to fly, do seem to result in longer lifespans [65,66]. Interestingly, certainly within mammals, the somatic mutation rate is dominated by an endogenous process and exhibits a strong negative correlation with lifespan, suggesting that they are evolutionarily constrained [67]. Plants also age, and while some only last a few weeks, some tree species can last 1000s of years. As in animals, without the purging effect of meiotic recombination, the accumulation of somatic mutations will eventually lead to mutational meltdown via a Muller’s ratchet mechanism. Often, their lifespan, like animals, is shortened by incidental problems, such as environmental change or infection. To overcome mutational meltdown, some have evolved very selective stem cell division patterns to minimise this effect [68]. One way of looking at this is that as the informational memory system becomes more damaged, the ability to maintain adaptive robustness slowly diminishes, ultimately leading to the organism dying because it can no longer maintain its structure.

It therefore appears that each species has a genetically determined lifespan, but that lifespan is highly modifiable by the environment. For instance, in humans, it has long been thought that genetics explains only about 15–30% of the variability, but it is possible that it could be as low as 10% [69]. Environment clearly has an impact. If *Caenorhabditis elegans* is exposed to ROS during embryogenesis, its lifespan is increased by invoking a protective response in the soma; this is in effect a hormetic mechanism which enhances its robustness and can be recapitulated by modifying mitochondrial function [70]. Even in the adult, damaging DNA in the germline can induce, via the innate immune system, the upregulation of the ubiquitin-proteasome system (UPS) that enhances proteostasis in the soma, making the organism more stress-resistant [71]—and could be viewed as a kind of “disposable soma in reverse” [72]. The key here is that the dose is important; low doses of genotoxic stressors, via the immune system, actually lead to improved soma homeostasis—however, chronic stress leads to inflammation and degradation [73]. This echoes the existence of a hormetic “goldilocks zone”.

Overall, the rate of ageing, and appearance of disease, is determined by genetics to some degree, and most obviously dictates lifespan for each species—and certainly for non-humans, it is balanced by predation and the environment. Humans are slightly different as they can extensively modify their environment and have managed to remove the threat from most predators, so are more likely to die of diseases of old age. What is clear is that ageing is associated with a progressive loss of resilience, and for humans, it does seem to predict human lifespan, which does seem to match what is observed with a theoretical limit between 120 and 150 years [74]. Thus, managing the stress response, it seems, is key in determining a healthy lifespan for most organisms and optimises their metabolic flight envelope.

### 2.6. Membranes and Ageing

Finally, there is perhaps a highly significant finding, and that is the relation between membrane unsaturation, and thus, propensity for peroxidation, antioxidant mechanisms and species longevity. As an organism ages, its membranes tend to lose fluidity, but this is despite an increase in unsaturated fatty acids, which fits with the increased oxidative stress. The increasing unsaturation is probably an attempt to maintain fluidity. Critically, long-lived species, like some birds and humans, have less powerful antioxidant systems compared to shorter-lived species like rats, but their membranes have much lower levels of unsaturated fatty acids. In effect, longevity is associated with a reduced propensity for lipid damage, which can also lead to damage to other systems. This is clearly very important in mitochondrial longevity and function [75].

There is perhaps a further hint here, and that is that there are data that mitochondria run at a higher temperature than the rest of the cell [76], which has prompted some to take mitochondrial control of temperature much more seriously because temperature modulates cellular functionality on so many levels [77]. This might suggest that not only are compounds like CBD likely to restore optimum fluidity by their physical interaction with membranes, as well as their antioxidant activity, they could also control temperature by modulation of uncoupling systems.

## 3. Phytocannabinoids, Mitochondria, Zeitgeist and the Lost Traveller

The previous discussion lays out the argument that we may have to view life from an adaptive thermodynamic viewpoint and its likely origins, which means we may also have to consider the problem of how phytocannabinoids work from the same perspective. This includes effects on ageing; dissipation and, thus potentially, fields; of course, the mitochondrion; and, very likely, all charged membranes. Defining how they work also depends on where they go in the cell, which is perhaps something that has not been clearly resolved yet; being lipophilic, compounds like THC or CBD could end up in most membranes.

Unfortunately, the beliefs and thinking of any period in history tend to drive where scientists look, and more often than not, this is reinforced as funding for researching ideas outside of this thinking is limited. In this light, however fascinating and important the endocannabinoid system is, it could be argued that it has been a bit of a “red herring” in terms of trying to understand how phytocannabinoids work. (The term “red herring” was coined by William Cobbet in 1807 in a story about using a smoked fish to distract hounds from chasing a rabbit [78].) It is thus interesting that we have again reached a point, as reflected in the zeitgeist of the time and discussed in depth in the 1980s [79], where the receptor versus membrane argument has resurfaced. The only clear consensus in the wider scientific literature is that phytocannabinoids are generally anti-inflammatory and lipophilic. Interestingly, there was an emerging paradigm in the 1980s about the importance of the phenolic group [79]; this may also turn out to be key. In this section, we briefly review these “zeitgeist” ideas and summarise what is known about the phytocannabinoids and mitochondrial function but also highlight some new thinking, for instance, around the chemiosmotic theory, which may also have a bearing on how these compounds work.

### 3.1. Zeitgeist and Challenging Dogma—The Not So Simple Electrical Mitochondrion

The science and beliefs of a time will often drive the thinking around an underlying hypothesis to explain an observation. For example, it has long been known that many secondary plant metabolites can be useful medicines, so research has focussed on how they might be working in humans, which means that a largely conventional pharmacological approach has been used. What they do in plants has perhaps been less of a priority. In turn, the “how they work” has been dependent on current definitions, causes and understanding of disease. Furthermore, the link between disease and ageing, although broadly accepted, has not been fully explored as we still do not fully understand the ageing process. Plus, as funding for research often follows the trends of the time, and thus, the current scientific zeitgeist, it can result in certain areas not being fully investigated—occasionally leading to important scientific leads not being followed up.

So, where should we have started when thinking about how phytocannabinoids work? As has often been said by a local asked by a lost traveller looking for directions: “I wouldn’t start from here”. We would argue that the obvious place is the origins of life, and thus what disease is, and then, of course, the plant. The problem here is that for many years, research, in terms of trying to understand how natural products work, has mainly centred on animal cells. And this includes mitochondria, even if they were studied initially in plants. Medicine has generally focussed on humans, and possibly their animal companions, but has not really embraced the origins of life, evolution and in particular the biology of plants, although the compounds they produced have been of great interest.

There is virtually no research studying the effects of these compounds on the mitochondria in the plants themselves, which mirrors plant mitochondrial research in general. For instance, although it has been known for some time that mitochondria are important in managing oxidative stress in plants, for many years, a lot less was known about their antioxidant systems compared to animal mitochondria [80]. However, it is now becoming clearer how, for instance, plants manage oxidative stress using uncoupling proteins (UCPs) and alternate oxidase (AOX), which can be viewed as a dissipating mechanism [5]. And, like animal cells, plant cells can also swap mitochondria [81], and during cold stress, they can fuse their mitochondria into reticular networks while increasing autophagy [82]. Furthermore, it is also becoming apparent that that just like animal cells, they communicate with each other, both in the short and long range, especially when stressed, using a variety of mechanisms, ranging from auxins, salicylic acid, ethylene, redox, extracellular vesicles (EVs) and other as yet unidentified methods. Plants therefore appear to have a mitokine-like system [83]. In short, although there are some differences, plant and animal mitochondria are essentially very similar and likely communicate with each other.

Interestingly, there is also building evidence that plants use a bioelectric signalling mechanism—the “electrome”—which is integrated with both ROS and calcium signalling and modulation of H^+^ ATPase; for instance, electrical signalling seems to play a role when a tomato fruit is attacked by a herbivore to transmit information to the shoots [84]. Of course, the idea that life has an “electrome” has been long suggested and goes back to the time when physics and biology were vying for ownership of the newly discovered electricity, as exemplified by the dual between Volta and Galvani in the 18th century [42]. It now seems that an injured leaf, damaged by herbivory or excess light, not only sends an electric signal around the plant enabling adaptation, but if the plant is touching another plant, this danger signal can be passed onto other plants, even different species, resulting in network-acquired acclimation. Interestingly, this signal is mediated by activity in ion channels, accompanied by waves of ROS and non-photochemical quenching (NPQ)—and the plant-to-plant signal can also be transmitted by a copper wire from one plant to another [85]. It has also been suggested that electrical signalling could also induce PCD [86]. As might be expected, the plant stress response can be induced by electrical stimulation [87].

As the plant stress response is integrated with mitochondrial function [88], with UCPs playing an important role, perhaps enhancing metabolic flexibility [89], it would seem likely that there is a strong link between bioelectric signalling and mitochondrial function—possibly implying that compounds like the phytocannabinoids could be modulating this system at multiple levels. Central to this process would be modulation of dissipation and electric fields, perhaps by influencing ATPase and ion channels, both at the cell surface and also at the mitochondrial level. Tellingly, the one protein involved in the bioelectrics of regeneration in planaria, Arabidopsis, deer and the axolotl is a key subunit in both gap junctions and the V-ATPase, ATP6V0C [55]. As adapting to stress requires energy, this would suggest strong coupling between mitochondria and cell surface components involved in signalling. This integration can be traced all the way back to the endosymbiotic event that led to the eukaryote, and if Morelli et al. are even partly correct about a new chemiosmotic theory suggesting that protons move along a membrane rather than through it [90], it puts membranes centre stage—and thus potentially, lipophilic compounds in modulating the process and, of course, calcium signalling. The key is that as indicated earlier, membranes can be viewed as thin capacitors [44]. In this light, it is thus interesting that another reason that Morelli suggests that the standard chemiosmotic theory may need revising is due the free energy barrier created by the electrostatic charge next to the membrane, which may effectively prohibit the free movement of aqueous hydrogen; it may exist as a hydronium ion. In effect, protons could not flow in the way originally suggested by Mitchell. This would explain why the F1 component of the ATPase and the arm of, say, complex 1, protrude more than 10 nm from the membrane [90]. Clearly, more research is needed, but the implications are fascinating.

This of course hints that we may need to rethink how uncoupling may operate. For example, it has now been shown that dinitrophenol (DNP), one of the most powerful uncoupling agents and protonophores known, although it can induce some uncoupling in a planar lipid bilayer, has a greatly increased rate in the presence of UCPs and, interestingly, with the adenine nucleotide translocase (ANT) [91]. Although there is not a huge amount of research, several bioactive plant compounds, including quercetin, resveratrol, ursolic acid, berberine and salicylate, as well as fluoride derivates of curcumin, are either direct uncoupling agents or induce UCP upregulation [92,93,94,95,96,97]. Uncoupling can also induce mitophagy, and many uncoupling agents also affect other membranes and organelles, including the lysosomal system [98]. Whether or not we may need to rethink the chemiosmotic theory from the stress response point of view, the manipulation of uncoupling would appear to be very important and an intrinsic function of plant secondary metabolites. This is of course dissipation.

### 3.2. Phytocannabinoids and Animal Mitochondria—Nothing New!

In the 1970s, many groups were looking at the effects of phytocannabinoids on mitochondrial function; for instance, one of the earliest investigations, published in 1971, suggested that THC could inhibit ATPase [99], and in 1972, a group provided data that it could uncouple state IV respiration of purified mitochondria, inducing them to swell, and that in micelle experiments, it could displace cardiolipin [100]. Around this time, Bino and colleagues also showed that THC could alter mitochondrial shape, dependent on the dose, which correlated with their respiratory rate [101]. In 1974, a paper was published indicating that THC distributed to both the nucleus and mitochondria in the liver, brain and kidney [102]. In a further set of experiments published a few years later, the same group confirmed that THC, after injection into live animals, rapidly appeared in the mitochondrial fraction of several tissues—within 20 min [103]. During this time, another group showed that THC could inhibit complex 1 and possibly complex III of the electron transport chain [104]. Interestingly, it was also shown that both CBD and THC could also inhibit a vesicle Mg^2+^ ATPase, with CBD being more potent; THC appeared to show a biphasic effect, stimulating at 1 µM and inhibiting above this. The authors thought this could explain the anti-epileptic actions of these compounds [105]. And then in 1985, another group confirmed that THC could induce mitochondrial swelling in several different tissues in rats [106]. Thus, the thinking at the time was very much in step with the general scientific interest in mitochondrial function and disease, and if the data were correct, both CBD and THC seemed to be directly affecting mitochondrial function. However, in 1988, Devane and colleagues published a paper demonstrating that they had identified a cannabinoid receptor in the brain [107], which led to the discovery of the endocannabinoid system and an explosion of research into both it and the phytocannabinoids [108].

Following this, publications on phytocannabinoids and mitochondria then seemed to totally disappear for a number of years; however, in the 2000s, there was a general resurgence in interest in mitochondria, in particular, around cancer. With it came a new swarth of papers suggesting that both THC and CBD could modulate mitochondrial function. For instance, THC could disrupt mitochondrial function, inhibit respiration and could result in cell death [109,110], and it could biphasically affect complex 1 function [111], while it could also restore mitochondrial function, possibly via activation of the peroxisomal proliferating activate receptor gamma (PPARγ), in a neuroblastoma cell line [112]. In an interesting twist, Fisar and colleagues also suggested that THC had both direct and receptor-mediated effects on mitochondrial complexes, supporting some evidence that mitochondria could contain a cannabinoid receptor (CBR), as well as indicating that CBD also affected the ETC: the IC_50_ in relation to respiration was 8.2 and 15.1 µM for CBD and THC, respectively [113]. This led to the idea that THC could disrupt cellular respiration to limit neuronal activity via mitochondrial CB-1R interacting with complex 1 [114] and, later, the publication of data indicating that its inhibitory effects on mitochondrial function and bioenergetics could have adverse effects during post-natal development [115]. In fact, it is now being suggested that mitochondrial CB-1Rs could be playing a much wider role in brain energy processes than previously thought [116]. Another group also showed that THC could disrupt neuronal cell mitochondrial function, affecting complexes I, II and III, decreasing coupling and enhancing ROS, suggesting that this could be important in cannabis-related strokes [117]. More recently, data have also been published showing that THC could induce mitochondrial dysfunction in placental cells, resulting in decreased ATP and increased ROS production, reducing membrane potential, but increased proton leak—and interestingly, increasing the levels of heat shock proteins, HSP60 and HSP70 [118]. In contrast, one group showed that THC was not directly toxic to cardiac mitochondria; indeed, it might even be protective [119]. We have also shown that phytocannabinoids can affect mitochondrial dynamics [10,120,121]. Very recently, Machado and colleagues, in a preprint, seemed to demonstrate that chronic administration of CBD to mice seemed to reduce the expression of ETC and TCA components in the hippocampus, which, as the authors pointed out, seemed to be in contrast to previous reports showing that lower doses or single administration increased mitochondrial complex activity. They concluded that CBD can have a biphasic effect on mitochondrial respiration [122].

As mitochondria are key in controlling PCD, that both THC and CBD can act as anti-cancer agents by inhibiting mitochondrial respiration in cancer cells is perhaps also relevant [123]. Indeed, the data from a pilot phase 1 clinical trial of THC in GBM patients were published in 2006 [124], so the idea is not new. Overall, the interest in phytocannabinoids as anti-cancer agents continues to grow, but with differences of opinion in precisely how they might be working, ranging from ECS-focussed for CBD, with little on mitochondria [125]; immunomodulatory, but nothing on mitochondria [126]; to more mitochondrially focussed thinking involving CBD [127]. In fact, a recent review also concluded that CBD can, and does, modulate mitochondrial function, but the range of effects, from neuroprotection to cancer cell killing, depend on dose, cell status and the type of cell—with several different targets, for instance, the voltage-dependent anion channel 1 (VDAC1), which could help to explain its role in CBD’s modulation of calcium homeostasis [128].

Overall, there is reasonable evidence that phytocannabinoids can modulate animal mitochondrial function, which is supported by good theoretical reasons, and it is likely that the outcome could be biphasic and dependent on cell state, dose and temporality. This would suggest that the modulation of mitochondrial function could play a role in their mode of action. Indeed, although much of the literature has focussed on the ECS as being involved in how they work, even here, a strong case can be made that this system also modulates mitochondrial function [129], suggesting that whichever way the mode of action is viewed, mitochondria are likely to be involved.

### 3.3. Phytocannabinoids and Plant Mitochondria

Unfortunately, compared to studies looking at compounds like THC and CBD on animal mitochondria, there is virtually nothing on what they might be doing to plant mitochondria in situ. The only data we have managed to find state that tetrahydrocannabinolic acid (THCA) and cannabichromenic acid (CBCA) can induce cell death of the leaf cells from the plant by inducing mitochondrial permeability transition (MPT) [130]. Further research confirmed that other phytocannabinoids, such as CBDA, could also induce cell death via MPT in cannabis leaf cells. The effect was repeated in isolated cannabis plant mitochondria, which exhibited swelling and a fall in mitochondrial potential—although the dose was quite high, at around 200 µM. The authors suggested that this was key necrotic mechanism—and fitted with the importance of programmed cell death in plants [131]. A key moiety in this is likely to be calcium, which we will discuss in more detail later. However, the high doses would fit with what is seen in animal studies (which even at comparatively much lower doses, can still induce cell death).

### 3.4. What Do Phytocannabinoids Do in the Plant—Lessons from Other Plant Compounds

Given the paucity of research on phytocannabinoids and mitochondria in plants, what can we infer from their suggested functions in plants and the role of other secondary plant metabolites (SPMs)? It is generally accepted that SPMs are involved in stress resistance, with plants increasing their production in response to numerous stressors, ranging from UV to drought, salt stress, herbivores, insects, pathogens and competition from other plants. As we have suggested, one way to view how they might work as medicines reflects their evolved ability to manage the dissipation of energy [10].

A key function of SPMs is managing oxidative stress and redox, indicating that they are hormetic—so, depending on context and dose, inducing a range of responses, ranging from proliferation to death [132]. Critically, as indicated earlier, mitochondria are important in the plant’s stress response [88,133]. Their ability to soak up free radicals is well described, acting as net sinks of ROS and hydrogen peroxide and, of course, via respiration which also removes oxygen, which itself could lead to free radicals [59,60,134]. Indeed, oxygen toxicity has been long recognised and managing it at the cellular level has been a constant challenge in evolution [135]; it is now thought that hyperoxia, for instance, can destabilise Fe-S-containing proteins, with the ETC being particularly vulnerable [136]. It is thus perhaps relevant that many phenolic plant compounds are well known to have direct antioxidant activity and many of them also modulate mitochondrial function in multiple ways [137]; it has also been suggested that many can also act as triplet state quenchers, with their delocalised pi electron cloud being key in their ability to dissipate energy [138]. It would seem entirely likely that these functions are linked through evolution. For example, being able to act as direct antioxidants, dissipate light energy and enhance mitochondrial function, both for energy production but also via uncoupling, are all useful stress resistance mechanisms.

So, what is the thinking about what phytocannabinoids do in the plant? Their precise role has been much debated, but like other plant secondary metabolites, the consensus is that they are involved in resisting stress, for instance, protection against UV; resistance to desiccation, herbivores and insects; as well as anti-bacterial and anti-fungal actions [139], the latter, as they can induce plant cell death [130,131], mirroring what happens in animals—for instance, in resistance to viruses. Their direct effects on bacteria have also been long observed [140,141]. More recently, and perhaps very significantly, it has been shown that CBD can induce depolarisation of the cytoplasmatic membrane in Gram-positive bacteria at concentrations of less than 1 µM [142]. THC has also been shown to have anti-viral activity [143], and very recently, CBD was found to inhibit SARs-CoV-2 replication by inducing host cell endoplasmic reticulum stress and innate immune responses [144]. We have also shown that CBD can modulate extracellular vesicle (EV) production both in animal and bacterial cells, which, because EVs are involved in both immunity and bacterial function [145,146], could provide insight to a mechanism of action. For example, certainly for their anti-bacterial actions, both membrane depolarisation and interference with quorum sensing may well be important.

## 4. Clues from Mitochondria and Calcium Signalling

Mitochondria are pivotal in calcium homeostasis and signalling [147], which can be traced back to the origins of eukaryotes [24]. Thus, the finding that CBD modulates multiple ion channels and calcium signalling [3] is perhaps very suggestive. It is well known that ageing is associated with rising inflammation coupled to decreasing mitochondrial function, epitomised by the term “inflammaging”. Recent data show that at least in macrophages, this coupling is strongly associated with a reduced ability of mitochondria to take up calcium, involving the mitochondrial calcium uniporter (MCU), and increased nuclear translocation of NF-κB. This supports the emerging role of mitochondria in calcium signalling and modulation of the immune response [148]. Although data also suggest that THC can also modulate calcium signalling in a non-CBR-dependent way, for instance, via T-type calcium channels, transient receptor potential (TRP) channels and Cav channels [149,150,151], we will focus on CBD because of its known effects on mitochondria. However, as THC does also appear to modulate mitochondrial function, it is also likely to affect calcium signalling in this way too.

A key observation is that calcium signalling in plants is just as important as it is in animals and involves all the organelles. In fact, the coordinated calcium-based stress response in plants is similar to animals, with many comparable ion channels and sensors [152]. Calcium signalling is also key in cancer, with evidence that it is diverted more towards the nucleus and away from the mitochondrion [153]. It is also key in inflammation [154], hinting that at least one mode of action in both the anti-inflammatory and anti-cancer action of CBD could involve the redirection of calcium.

### 4.1. The Concentration Problem

There is perhaps an initial concept to consider, and that is the problem of concentration. As these compounds are highly lipophilic, then they will preferentially dissolve in membranes. Although this provides a whole new layer of modulatory possibilities, it tends to throw the conventional concepts of pharmacological activity and dose into disarray and certainly would help to explain the huge discrepancies seen in the literature about doses and effects. It is therefore perhaps necessary not just to think about the molarity of a phytocannabinoid solution added to an experiment but also the number of molecules per cell and the total lipid/membrane capacity of the cell to absorb these molecules. CBD, for instance, is virtually insoluble in water but readily dissolves in ethanol and has a log n-octanol-water partition coefficient (K_OW_) of about 8, which is why a lot of research has looked into how to deliver it as a medicine in terms of formulation [155]. However, as far as the authors can ascertain, there is no published research as yet that has studied its distribution in live cells—in particular, over time. In contrast, a few groups have apparently shown, following extraction of cellular components, that phytocannabinoids can be found in mitochondrial fractions (e.g., [102,156]). However, differential centrifugation of organelles is renowned for cross-contamination of lipids, so this needs to be interpreted with caution.

### 4.2. CBD Membrane Targeting and Calcium Signalling

It has been suggested that, teleologically, a key function of mitochondrial calcium uptake is to modulate mitochondrial metabolism [147]. In terms of the history of life, calcium signalling evolved in prokaryotes [157,158] and probably was key in how the original endosymbiotic metabolism evolved when eukaryotes came into being—the ancestors of the mitochondrion required calcium as much as their host [24].

A key point about any theory on how a drug may work will depend on where it reaches in the cell. As indicated, CBD is a highly lipophilic molecule and is known to dissolve in membranes, in particular, lipid rafts [159], and seems to preferentially insert in the hydrophobic region of the membrane, altering fluidity and, for instance, inhibiting sodium channel function [160,161]. This suggests, at least in animals, that CBD’s first port of call would be the plasma membrane. Data show that it can modulate the transient receptor potential vanilloid 1 (TRPV1) channel, which can cause an influx of calcium into the cell [162].

It is well described that mitochondria play a vital role in modulating TRPV1 signalling as the mitochondrial calcium uniporter (MCU) and the sodium/calcium exchanger (NCLX) are involved in this process [163]. Indeed, TRPV1 channels, as well as other TRP channels, act as sensors of noxious stimuli, and are part of the contiguous connection between the plasma membrane, the ER and mitochondria [164]. In terms of compartmentalisation, mitochondria and the ER are in close contact via mitochondrial-associated membrane (MAM) complexes, which transport metabolites, including lipids and signalling moieties, such as calcium [165]. As an example, it seems that activation of TRPV1 in muscle by high temperatures may well be important in stimulating mitochondrial biogenesis [166], which suggests how various stressors can upregulate metabolism. In short, TRPV1 channels are key in hormesis.

To date, several groups have shown that CBD can modulate animal mitochondrial calcium levels [167,168,169], with one possible target being VDAC1 [156]. Interestingly, Ryan and colleagues did seem to suggest it might be interacting with NCLX [167]. There is also evidence that both CBD and THC can be transported by fatty-acid-binding proteins intracellularly [170].

Unfortunately, apart from the Rimmerman data, there are no in-depth studies on intracellular location. But there are other studies with other plant compounds that might hint at a mechanism, for instance, resveratrol, which can directly target mitochondria [171], could be taken up into the interior of cells by a lipid raft-based endocytic mechanism [172], and edelfosine, which can accumulate in the mitochondrion, might also be taken by cholesterol-rich lipid rafts [173]. In short, they could certainly be transported from the plasma membrane to other organelles, for instance, if the lipid raft undergoes endocytosis. Given the lipophilicity of these compounds, it is thus highly likely that they will modulate membrane structure and thus the activity of ion channels.

### 4.3. Calcium Flux and Mitochondrial Dynamics Are Intimately Coupled

In previous sections, we reviewed data that show that not only can phytocannabinoids alter bioenergetics but that they also modulate mitochondrial dynamics. Indeed, changes in mitochondrial dynamics are linked with the stress response, ranging from fusion to the formation of toroids and complete fission. Mitochondria fuse to enhance oxidative phosphorylation by sharing mtDNA and other components; it may also be a mechanism for enhanced intracellular signalling and resistance to fission [174]. As stress increases, they start to detach from the cytoskeleton and form small toroids, or donuts, another stress resistance characteristic [175]. This is something we have observed with CBD [120].

Mitochondrial dynamics are therefore a very good way to study the stress response and, in particular, the fate of the cell and role in disease [176]. Of particular relevance is the fact that inflammation inhibits mitochondrial function and induces their fission—which is an adaptive response [177,178,179]. When linked to the relationship between mitochondrial dynamics and spatiotemporal calcium signalling [147], the ability of phytocannabinoids to modulate both calcium signalling and mitochondrial dynamics is perhaps defining.

In 2009, Duncan Ryan and colleagues showed that CBD, at 1 µM, could modulate cytosolic calcium concentrations bidirectionally in glial and neuronal cells. It appeared to involve CBD acting directly on the mitochondrion to control calcium flux; the direction, in or out, was dependent on whether the cells were in a hyper-excitable state or not, respectively. Their conclusion was that this was at least partly dependent on the ability of CBD to keep the inner mitochondrial membrane (IMM) NCLX in a confirmation that allowed it to pass these ions more freely. They suggested that under normal conditions, CBD induced the release of calcium from the mitochondrion (and into the cytosol), but in excitable conditions, with high levels of calcium coming into the cell, the mitochondrion effectively acted as a sink for excess calcium. In some of their experiments, they also showed that there was an initial biphasic effect on mitochondrial calcium, where calcium levels initially increased then decreased [167]. In this respect, CBD seemed to modulate mitochondrial calcium signalling resulting in a context-dependent restoration of homeostasis.

Later, Rimmerman and colleagues discovered that CBD apparently collocated with VDAC1. They showed, using BV-2 microglial cells, that CBD at 10 µM induced mitochondrial swelling, loss of membrane potential and increased ROS production. It could also induce an increase in intracellular calcium within 100 s; the levels then fell but rose again after 80 min. They also showed that CBD reduced the conductance of purified VDAC1 mixed with artificial membranes. They suggested that CBD reduced conductance at all voltages, and their interpretation was that this completely inhibited VDAC1 function in the whole cell, which resulted in cell death. However, they also made the point that CBD might also cause mitochondrial calcium overload due to the VDAC1 reduced conductance favouring the uptake of cytosolic calcium and activating the mitochondrial permeability transition pore (MPTP) [156].

It therefore seemed that both groups showed an initial intracellular spike in calcium on CBD treatment, although Ryan’s group looked at later time points, who also showed that in their cells, cyclosporin did not affect CBD’s actions, but an inhibitor of the NCLX channel did. In contrast, Rimmerman’s group showed the complete opposite. We suspect that part of this difference may be due to the different concentrations these groups used: Ryan used 1 µM, whereas Rimmerman’s groups used 5 and 10 µM. The dose is clearly important.

In another piece of research, Mato and colleagues also showed that CBD could induce a dose-dependent increase in intracellular calcium but in oligodendroglial cells. This was associated with a biphasic effect on mitochondrial polarisation (hyper-, then hypo-polarisation at 10 µM) and a dose-related loss in cell viability and then cell death (via both caspase-dependent and -independent pathways). They also showed a dose-dependent increase in ROS (increasing across the range of 100 nM, 10 and 100 µM). Interestingly, some of the toxic effect was reduced by reducing the extracellular concentration of calcium, suggesting that calcium influx into the cell was important, perhaps suggesting a role for store-operated calcium entry (SOCE). The effect of CBD was also inhibited by carbonyl cyanide-p-trifluoromethoxyphenylhydrazone (FCCP), which uncouples mitochondrial oxidative phosphorylation and decreases membrane potential, as well as by application of Trolox, which mops up free radicals. In addition, the loss of viability was not affected by antagonists of CB1/2, adenosine, peroxisome proliferator-activated receptors gamma (PPARγ) or vanilloid channels—ruling out most of the conventional receptors that have been of interest with the phytocannabinoids. Interestingly, they noted that the increase in ROS was commensurate with the increase in intracellular calcium. Overall they concluded that the CBD-induced increase in intracellular calcium was related to mitochondrial dysfunction [168].

### 4.4. Interpreting the Phytocannabinoid Effects on Calcium Flux and Mitochondrial Dynamics

There is a clear relationship between calcium flux and mitochondrial structure, but how does a single signalling system differentially control so many cellular functions? One clue comes from the fact that each transcription factor requires different oscillatory frequencies to become activated or inactivated; calcium nuclear signalling is pivotal, for instance, in neuronal functioning [180]. Critically, subtle imbalances in calcium signalling, due to an imbalance in adaptive homeostatic systems, are involved in many disease pathologies. Cell surface receptors generate calcium-mobilising secondary messengers, such as InP3 (inositol 1,4,5-triphosphate) which can release calcium from ER stores, or voltage-operated channels (VOCs) that can allow extracellular calcium in, or by releasing it from internal organelles. They are usually activated by calcium itself, which enables globalised calcium waves to propagate. For this to work, intracellular calcium is kept at very low levels by a mix of high-capacity, low-affinity pumps such as NSLX and low-capacity, high-affinity pumps, such as sarcoplasmic/ER calcium ATPase (SERCA). The system is further modulated by the presence of calcium-binding buffer proteins, that either attenuate the signal in the cytosol or store calcium in the ER, or by mitochondria. In turn, many proteins act as calcium sensors to detect the signal, including calcium/calmodulin-dependent protein kinases (CaMK), cAMP and mitogen-activated protein kinase (MAPK). Two important calcium-sensitive transcription factors are the cAMP-response element binding protein (CREB) and the nuclear factor of activated T cells (NFAT). In many diseases, the calcium signalling pathways often remodel and result in inappropriate signalling. For example, InsP3 signalling becomes elevated, resulting in excessive nuclear calcium signalling cardiac hypertrophy, leading to chronic heart failure. It is also thought that calcium dysfunction is important in schizophrenia and bipolar, as well as Alzheimer’s [181]. Both a fall in membrane potential and increased calcium efflux, as well as ROS, can activate mitochondrial nuclear signalling and induce mitophagy, so inducing a healthier population of mitochondria [182,183]. A critical point to remember is that for every 14 mV drop in mitochondrial potential, the ability to maintain the ATP/ADP ratio drops by 10 fold [184].

So, what might CBD be doing? In the first instance, it could activate something like a TRPV1 channel, which would lead to an increase in ER calcium, then bind to VDAC1, possibly reducing VDAC1 conductance to metabolites, but favouring calcium entry and stimulating mitochondrial function. It could then also perhaps inhibit ETC function, leading to a further specific ROS signal at complex 1, say, by inducing reverse electron transport. It could also then inhibit NCLX, leading to further calcium build up inside the mitochondrion. This would enhance ATP production and ROS generation, perhaps leading to membrane hyperpolarisation, but continued influx would eventually lead to reduced energy production and thus a reduction in mitochondrial membrane potential—a major driver of calcium influx, and, in effect, the system would self-limit. When permissive channels are open, calcium uptake by the mitochondrion is entirely dependent on its membrane potential and the principle of mass action. These would be highly specific adaptive signals, especially if it also changes membrane structure and, say, enhances hydrogen peroxide production. In the early stages, this suggests a positive feedback loop, but then as the mitochondrion becomes overloaded, it suggests a negative feedback mechanism.

Thus, the dose of the CBD is important, as well as the rate it diffuses through the cell and the metabolic state of the cell (for instance, in which direction the TCA cycle is running and whether it is predominately using glycolysis or oxidative phosphorylation). At a very early stage, or a low concentration, this intake of calcium might induce fusion and mild tubulisation—which might be dependent on the type of cell. This could be related to a VDAC1 delivery of calcium to the ryanodine receptor 1 (RyR1) channel, which would open first, but might not involve MCU1. As calcium influx continued, the dynamin-related-protein 1 (Drp1) [185] might start to become activated, initiating fission and then donuts. Donuts or toroids appear to be a stress-resistant form that can maintain oxidative phosphorylation [175]. At this stage, the mitochondria would still be very active and capable of storing extra calcium, and thus might stop the propagation of the calcium signal. However, as intra-mitochondrial calcium concentration reaches a critical level and metabolite flux is inhibited, mitochondrial depolarisation must start to occur. This could, at least initially, be related to MPTP opening and might well lead to mitochondrial swelling.

The MPTP can act as a kind of safety valve, flickering between an open and closed state, which temporarily reduces mitochondrial membrane potential and induces ROS, and possibly venting excess calcium. A little bit more stress may activate autophagy, then apoptosis, while excessive opening will induce rupture and necrosis. In effect, a whole range of responses may occur, ranging from adaptation to inflammation [186]. For example, high calcium flux through VDAC1 might push the cell towards apoptosis by overloading the mitochondrion with calcium. However, a degree of downregulation of VDAC1 inhibits mitochondrial activation of the inflammasome and propagation of ROS [187]. In effect, by reducing calcium flow into the mitochondrion, the cell might be induced towards mitophagy rather than apoptosis and would be associated with reduced inflammatory signalling.

The formation of the NLRP3 inflammasome is also highly dependent on calcium [154]. In this regard, it is perhaps relevant that that oxidised mtDNA might exit the mitochondrion via the MPTP and VDAC dependent channels to activate the neuronal NLR-family pyrin domain-containing protein 3 (NLRP3) inflammasome [188]. An interesting parallel exists here with plant immunity: activation by a pathogen of the nucleotide-binding leucine-rich repeat (NLR) receptors can activate effector-triggered immunity (ETI). During this activation, an increase in ATP is needed to enable oligomerisation of NLR to form the resistosome, which triggers calcium entry and cell death. In animals, a similar rise in ATP, generated by mitochondria, is required for the NLR to form the inflammasome—potentially forming an amplification loop [189]. If CBD is modulating VDAC1, this could be a key mechanism controlling activation of the NLRP3 inflammasome. Interestingly, data are also building that fumarate overload in mitochondria can lead to changes in mitochondrial morphology and the release of mitochondrially derived vesicles (MDVs), which contain mtDNA that activate inflammatory pathways; this pathway is independent of the VDAC one [190]. This might suggest that CBD could have a much more nuanced control of inflammation. Others have also suggested that CBD could be modulating the inflammasome, which could be key in how it might help in neuroinflammation and stress-related psychiatric conditions [191].

These ideas could explain the Ryan data [167]: in normal conditions, 1 µM CBD induces mild mitochondrial hyperpolarisation and increases intra-mitochondrial calcium, which is then slowly released back into the cytosol for recycling. In hyper-excited cells, the increase in mitochondrial membrane potential might induce the flow of calcium into the mitochondrion from the cytosol. In this scenario, NSLX was active (and could be inhibited) but the MPTP was not (and thus inhibiting it had no effect). In contrast, Rimmerman used higher concentrations of CBD [156]; in this case, cyclosporine did have an effect, suggesting that the mitochondria were becoming overloaded (and were swelling), because it reduced MPTP activity (amongst many targets, it can bind the MPTP cyclophilin D, inhibiting its function [192]). Both of these scenarios are supported by our data and that of Mato [168], who showed that at 1 µM, CBD increased mitochondrial membrane potential, but did not appear to inhibit it. In contrast, at higher concentrations, the effect was clearly biphasic, with first an increase then a decrease in potential. This would suggest that there is a concentration-dependent effect of CBD as it diffused through the cell and into different cellular structures.

The key point here is that the influx of calcium into the mitochondrion is a key stress response, both in plants and animals, which raises the question—do these compounds, if given at high enough doses, induce inflammation? Interestingly, there is little emphasis on the inflammatory effects of these compounds, but a lot on their anti-inflammatory effects—all of which is attempted to be explained by their apparent modulation of the more accepted targets, such as CB1 and CB2 receptors—a very endocannabinoid viewpoint [193]. However, a recent review looking at in vivo studies, although showing that in general they could lower inflammatory and increase anti-inflammatory cytokines, did report that in some cases they were not effective, especially at higher doses, and a few studies even showed an inflammatory effect. Overall, CBD seemed have better anti-inflammatory effects than THC, but authors of the review indicated that there is actually quite limited data and a great deal more research maybe needed when moving to clinical trials [194]. However, it has now been shown that vaping CBD can actually induce lung inflammation, tellingly, more so than vaping nicotine, with data indicating it induced neutrophil apoptosis and increased elastase levels [195]. In this regard, there is good evidence that as nicotine modulates mitochondrial function [196] and CBD can inhibit the metabolism of nicotine [197], there could certainly be some kind of combined effect on mitochondrial function. The conclusion could be that compounds like CBD could be mostly anti-inflammatory, but induction of rapid cell death could result in inflammation in some circumstances.

One of the rather intriguing possibilities is that mitochondrial fragmentation could be a negative feedback mechanism to control excessive calcium signalling. It might explain why inflammation is associated with fragmentation and then the induction of autophagy; mitochondrial fragmentation is the beginning of an anti-inflammatory feedback system. Thus, in effect, CBD in particular may induce a negative feedback mechanism that restricts excessive inflammatory calcium signalling, part of which likely involves activation of the stress response system involving Nrf2 and suppressing activation of the NFAT, and likely, the inflammasome.

**Figure 1:**
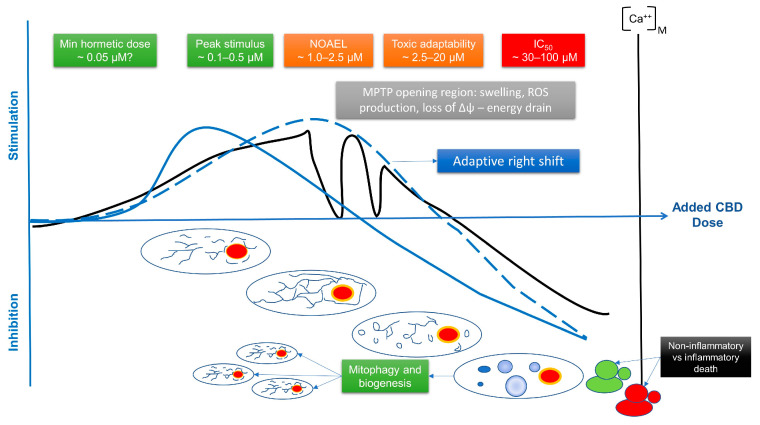
Predicted biphasic effect on mitochondrial calcium and dynamics, based on our own published and unpublished data and that in the literature. The key point here is that an initial calcium burst into the mitochondrion would stimulate its function, but as the dose increased (and the compounds spread through the cell), it would have the opposite effect. This would effectively buffer the mitochondrion from excitatory/inflammatory signals from the rest of the cell, while activating the well-described mitochondrial nuclear retrograde cytoprotective response. Critically, the response would be dictated by the metabolic status of the cell, its mitochondrial capacity, the direction the TCA cycle was running in, so anabolic or catabolic, and thus the balance between oxidative phosphorylation and glycolysis, and its state of oxidative stress. NOAEL, no observed adverse effect level.

As indicated, a key component of the calcium flux from the ER to the mitochondrion are mitochondrially associated membranes (MAMs), which are a kind of membrane-based complex that connects mitochondria to the ER that transports many metabolites, including lipids. They have many functions, such as controlling the flow of calcium to control cell death in cancer [153] or in exercise preconditioning that protects the heart [198]. Another pathway that is also potentially important is the one involving the mitochondrial anti-viral signalling protein (MAVS) and modulation of the inflammasome [199]. Finally, there is some interesting emerging evidence that the sarcoendoplasmic reticulum calcium ATPase (SERCA), which pumps calcium back into the ER by using ATP, could be part of a futile cycling/uncoupling mechanism to generate heat [200,201]. Again, this might indicate that activation of mitochondrial function and the movement of calcium could have a number of effects, but one of them could be to encourage a form of uncoupling—dissipation. Figure 1 summarises how dose could affect mitochondrial dynamics in relation to calcium signalling.

### 4.5. Cannabidiol and Subtle Mitochondrial Manipulation: Hexokinase, the Warburg Effect, Apoptosis and Sub-Lethal Adaption 

It is well known that calcium, apoptosis and mitochondrial function are all linked. As is becoming clear, the mitochondria, and the apoptotic machinery and proteins like the caspases and the Bcl system, seem to control a whole range of responses, ranging from mild adaptation to suppression of inflammation, to apoptosis, to inflammatory pyroptosis—the concept of sub-lethal signalling. In effect, mitochondrial outer membrane permeabilization (MOMP) is not always lethal and leads to a whole range of outcomes. When it goes wrong, it can lead to chronic inflammation. The origins of this system stem from the role of the mitochondrion in pathogen resistance, and likely, the original endosymbiotic event that led to the modern cell. Critically, quite apart from its role in metabolism, VDAC1 can also form pores, leading to release of mtDNA and activation of inflammation [202,203]. Thus, modulation of VDAC1 by compounds like CBD could modulate and potentially inhibit this process.

But there are other VDAC-related responses that CBD could modulate. For example, the binding of the enzyme hexokinase (HK) to VDAC1 activates it and results in the phosphorylation of glucose to G-6-P, the rate-limiting step in glycolysis, as well as putting it near the mitochondrial source of ATP. The binding of HK to VDAC1 reduces its conductance and inhibits the binding of apoptotic factors, such as Bax, or the release of cytochrome C; it also suppresses the release of ROS via VDAC1, reducing ROS-activated cytosolic apoptosis. Hence, by binding to VDAC1, it enhances cellular survival. However, reducing VDAC1 activity can also lead to an increase in internal mitochondrial ROS that can activate the MPTP. Dissociating VDAC from HK, as well as inhibiting VDAC, is thus a therapeutic approach in cancer treatment, and reducing its activity may therefore be beneficial [204]. Data are suggesting that the binding of HK to the outer mitochondrial membrane (OMM) stabilises it, maintaining mitochondrial membrane potential and prevents Bak and Bax binding, so inhibiting TNFα-induced apoptosis [205]. In this regard, we have previously suggested that by binding to VDAC1, CBD may not only reduce its conductance but also inhibit HK (for instance by causing it to dissociate), which would reduce the capacity of the cell to undergo glycolysis, forcing it to utilise respiration. This would have both a profound anti-cancer and an anti-inflammatory effect. In short, it could have an anti-Warburg action [121]; for many oncologists, this is thought to be a key outcome for cancer treatment. However, as is now being realized, cancer cells are still highly dependent on mitochondrial function, even if glucose oxidation is reduced. The mitochondrial phenotype just seems to change according to need [206]. It is thus relevant that a recent publication has not only confirmed that CBD binds to VDAC1, but that it is likely that its anti-prostate cancer activity could involve modulation of HKII activity. Interestingly, this was associated with an enhancement of glycolysis, reduced respiration and ATP production, enhanced mitochondrial fragmentation and autophagy, and a loss in mitochondrial mass at CBD concentrations of less than 10 µM. The addition of CBG enhanced the anti-cancer effect, although on its own, it induced a slightly different metabolic profile [207].

### 4.6. A Calcium Mode of Action Viewpoint

We can now outline a more conventional integrated mode of action. In effect, low-dose phytocannabinoids may activate an early plasma membrane-based sensor mechanism represented by a number of channels, such as TRPV1, other receptors, and pathways via modulation of membrane thermodynamics leading to charge/redox changes (such as the ERK pathway and calcium flux). The molecules themselves could then potentially disseminate in the internal membranes, working their way through the ER and into the mitochondrion, and quite possibly, on towards the nucleus. The response of the cell is clearly part of a hormetic process (due to the adapt or be removed principle), and as well as the mechanisms described above, it would also activate the classical stress/xenobiotic response [22,208]. In this respect, modulation of membrane fluidity and associated proteins is an important way of both detecting stress, signalling it, as well as inducing adaptation.

As we suggested before, with regards calcium, we now have the interesting possibility that CBD, at least, may initially induce mitochondrial uptake of calcium, but then reduce it via inhibiting VDAC1 located at the MAM, which would be linked to the inner mitochondrial membrane mitochondrial calcium uniporter (MCU). It would also reduce the mitochondrial membrane potential. Interestingly, the MCU plays a critical role in controlling excitotoxicity by directly channelling calcium into the mitochondrion from the NMDA receptor; its downregulation protects the mitochondrion from overload, preventing oxidative stress and mitochondrially induced apoptosis [209]. In effect, there is a clear physiological precedent related to mitochondrial inhibition in controlling cell fate by not taking up calcium.

In line with this, another observation is that glutamate also modulates VDAC function [204]. This coupled with the possibility that CBD might inhibit HK function, while possibly, at least initially, enabling VDAC1/NSLX efflux, could also act to reduce excessive stress on the mitochondrion. This might be further enhanced by perhaps modulating MPTP function via the proposed interaction of the pore with VDAC. Another potential possibility is suggested by the observation that VDAC1 plays a role in the conduction of ROS generated from complex 1 [210]; this would suggest that inhibiting its function might reduce excessive cytosolic ROS signalling. However, these compounds have also potentially been shown to directly modulate the ETC [111,113], which could be key in how they result in different effects in different cells by modulation of ROS from different complexes. For instance, there is some evidence of a synergistic effect of THC and CBD in inhibiting respiration by downregulating respiratory chain proteins in glioblastoma cells [123], while CBD has been shown to increase the mitochondrial complex and creatine kinase activity in rat brains [211].

The overall effect therefore would be to attenuate high-amplitude cellular signalling into the mitochondria, which would reduce the chances of mitochondrial overload and thus activation of the classical activating signalling pathway. Critically, at the same time, it would trigger a more controlled adaptive retrograde signalling response involving mitophagy and biogenesis. The simplest way to view this is that initial interaction with the cell would activate xenobiotic sensor pathways that would activate mitochondrial function by increasing calcium flux into it. However, as levels rose, mitochondrial function would become inhibited, depolarising it and decreasing levels of acetyl CoA, ATP and NADH, and might therefore induce mild ROS production, all of which would activate sirtuins and AMPK—the sirtuins are an ancient group of proteins involved in stress resistance [212]. The depolarisation would also reduce calcium uptake.

**Figure 2:**
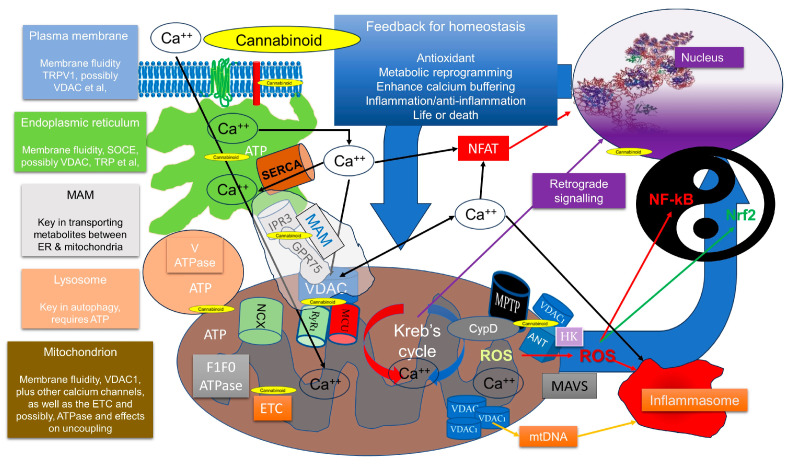
Summary of key factors related to calcium flux and mitochondrial control, and potential effect of CBD. However, it is possible that other lipophilic cannabinoids could also behave in similar ways. If CBD dissolves and concentrates in membranes, it is likely to affect the plasma membrane first, and then with time, potentially work its way into the mitochondria, probably via the MAM complex. It is likely to affect membrane fluidity and packing, and thus alter the confirmation of imbedded proteins, such as ion channels, receptors and electron transport chains, as well as modulate local redox. It is also possible that many of the imbedded proteins have evolved to be sensitive to potentially disruptive compounds like CBD and are thus part of the xenobiotic system. Any alteration in membrane structure will elicit a homeodynamic adaptive response, with calcium flux being a key one, which is coupled to redox and bioenergetics and thus mitochondrial function. Because mitochondria are central to inflammatory and calcium signalling, their prior health and metabolic state will determine the outcome. For example, dysfunctional mitochondria will have less capacity to produce ATP and thus result in inefficient autophagy, will produce more ROS and will not be able to buffer as much calcium, potentially leading to a feedforward loop, but if mitochondrial turnover and replacement are stimulated, they will be able to control their redox status better.

This reduction in energy output is well known to alter chromatin structure, invoking a cellular phenotype that upregulates mitochondrial biogenesis, reduces oxidative stress and suppresses inflammation [213]. The effects on intracellular and intramitochondrial calcium could therefore be informative, as indicated in figure, which superimposes the biphasic response curve and a possible effect of CBD on mitochondrial calcium concentration. Of particular importance is where calcium from the ER ends up with different doses of these compounds. It is now thought that if calcium flux into the mitochondria is inhibited, there is an outflow into the cytosol locally, which activates autophagy [214]. Autophagy is a key component of inflammatory resolution. However, blocking mitochondrial uptake might also induce a localised rise in calcium which might also inhibit calcium channels. For instance, TRPV1 is well known to display rapid desensitisation/tachyphylaxis by a number of mechanisms, including one involving calcium and calmodulin; in contrast, it can be sensitised by ATP. It is also expressed in the ER itself [215]. Figure 2 summarises the key components.

## 5. Beyond the Conventional

In this paper, we take the “origins of life explains extant biology” approach to outline another way to think about how phytocannabinoids might be working as medicines and, perhaps, to move from conjecture towards a more solid theory. The central point is that all life is based on generating a membrane potential that likely has its origins in fulfilling entropy’s drive to equilibrium and is explainable from both quantum mechanics and adaptive thermodynamics, which can only occur by membranes having the right level of fluidity and structure, but this has to resist potentially damaging redox shifts. This leads to the concept of life as a self-organising, far from equilibrium reproducing dissipative structure, where the movement of charge results in life being electric in its operation. In this section, we bring several apparently disparate ideas together to provide a connected overview of the centrality of membranes in thinking how compounds like CBD may be working. For example, it could modulate dissipation as an adaptive response via calcium flux and could alter bioelectrics.

A key concept to bear in mind here is that as homeostatic signalling complexity increased during evolution, it would have been built on simpler and older systems, which themselves would be based on prebiotic molecules and ions. Today, this means that entire complex systems are built around the control and movement of ions like calcium. Other classic examples of this are the iron-sulphur proteins and aromatic cofactors involved in electron and proton transport/charge separation and course, membranes. It may well be possible to say the same thing about secondary plant metabolites, which would have evolved from some basic structure; this would imply that they do not interact with a single protein or pathway, but many—as these systems evolved around them to amplify and control a fundamental principle, not the other way around. This might suggest we should talk about “prefactors” not “cofactors”.

Hence, with compounds like CBD, we cannot apply the classical pharmacology approach of “one compound, one target”, but that metabolism has evolved around some key property of this compound to ensure homeostasis. At its simplest, all life is based around charge separation and dissipation of energy, where the membrane is pivotal. So, the broad use of phenolic lipophilic compounds by plants to adapt to stress suggests something very fundamental about the way they work in the manipulation of dissipation and membranes—especially if they are involved in electron and proton transfer.

One emerging theory around the origin of pathology is the concept of “chemiexcitation”; it is well known that light can excite an electron to a higher unoccupied orbital, perhaps leading to a triplet, for instance, from exposure of chromophoric molecules in cells to UV light. However, a similar process can occur through normal metabolism, for instance, during inflammation. How and where this excess energy is dissipated is thus key in whether it is damaging. Many antioxidants, including plant secondary metabolites, have the ability to both prevent and quench triplet states due to their quantum structure [17,138]. The key point about this is that control of electron transfer and dissipation of energy can be a double-edged sword—at the right level and in the right place it can be protective, but it could also hinder both signalling and potentially, energy production. But, as mentioned in the introduction, and exemplified by the role of vibronic coupling in the ability of photosynthetic organisms to both be highly efficient in capturing energy, as well as dispersing it during photoprotection if it gets too excessive [11], we may also need to think quantum mechanically. What is it about many secondary plant metabolites, including phytocannabinoids, that helps plants survive stress and animals deal with illness? How can they kill one cell type, while protecting another? 

### 5.1. Dissipation in and out of the Goldilocks Zone—Controlling Life and Death

From the dissipative point of view, both prokaryotes and modern cells are constantly dissipating energy, with apparently 20–50% simply being “wasted”, for instance, by uncoupling in the mitochondrion or being dissipated across a bacterial membrane [216,217]. When viewed from the thermodynamic perspective, this is of course not being wasted but is essential to maintain structure. In fact, life is full of energy dissipating (“futile”) cycles. For example, it has been used to explain thermogenesis, for instance, UCPs and lipids, or pyruvate which may also control redox signalling [218]. As mentioned in the last section, new theories are also suggesting that calcium cycling could also be a thermogenic mechanism [200]. Another way to view it is that futile cycling is a price paid for fine control of metabolism [219]. Indeed, the link between uncoupling and lifespan has been investigated, and it is complex, but there is evidence that it can enhance lifespan in some models [220]. However, when one considers that the energy is being extracted primarily from the flow of electrons and protons, then clearly redox homeostasis is pivotal—because if this flow is not optimal or redirected, it can rapidly damage the system, involving reductive or oxidative stress, or perhaps, inhibit energy production. Dissipation is a good way to reduce oxidative stress, but the flip side is that it can also limit energy production.

As indicated, many plant compounds, in particular those containing phenol, can uncouple, for instance, salicylic acid, which is not only a key plant hormone but can also be used as a sunscreen—which may hint that they evolved to help maintain dissipation under stress. However, they could also be used to induce too much dissipation, which could explain, for instance, their anti-pathogen activity [10]. If the dissipative theory about life is correct, it might suggest that enhancing futile cycling could make an organism more adaptive and perhaps better at the fine tuning of homeostasis under stress, including inducing dyshomeostasis in its own cells or those of invading pathogens. Although not proven, it could be argued that compounds like CBD could induce a form of dissipation, which in itself could be key in a stress response, keeping the host organism operating in its Goldilocks zone, but it could also be used to take, say, a pathogen outside of its zone. In short, enhancing dissipation can be protective, but only up to a point.

### 5.2. The Host Xenobiotic Response 

The ability of a compound to excessively enhance dissipation and/or influence electron transfer could certainly be damaging. Think short circuiting. As indicated, CBD and THC are highly lipophilic but also contain a phenolic group, which is key in their clinical properties [79]. The phenol group, because of its delocalised electrons, has some useful antioxidant properties. Many plant-derived phenolic compounds can act as hydrogen donors and can end up as a more stable free radical or chelate metal ions, although they can also then act as prooxidants because of this. They can also strongly interact with proteins and affect redox cycles [221]. Interestingly, it is now thought that one of the reasons that plant compounds can be beneficial in animals is that at low concentrations their main mode of action may involve the generation of hydrogen peroxide—and activating the Nrf2 (NF-E2-related factor 2) axis [222]. Both CBD and THC can induce a stress response that involves activation of Nrf2 and suppression of NFkB [223,224,225], which does suggest that they induce a potentially similar mechanism.

In light of this, although it seems that animals have co-evolved detoxification systems to deal with the myriad of potentially toxic compound found in plants, its activation can also be beneficial, which has given rise to the concept of “xenohormesis”—whereby the animal is also using these compounds as an adaptive stress signal [226]. Mitochondria are thought to be central in initiating the hormetic response via “mitohormesis” due to sublethal stress [52]. Certainly, many bioactive compounds rapidly distribute to mitochondria in whole cells—quercetin and berberine being prime examples [227,228]. As indicated, many can uncouple oxidative phosphorylation, such as salicylic acid, with the phenol moiety playing a key role in this [96,229], while some, such as capsaicin, can activate TRPV1 and inhibit mitochondrial function [230]. However, the outcome likely depends on concentration.

This supports the hypothesis that many endogenous receptors and channels have coevolved to detect the presence of xenobiotics and activate detoxification pathways. This is epitomised by components such as cytochrome P450 and, in particular, Nrf2. Nrf2 is a redox-sensing transcription factor critical in both detecting ROS and xenobiotics and activates the phase II response and upregulates antioxidant systems. It is also a key component in the adaptation to ROS induced by increased mitochondrial respiration during calorie restriction and that induced by the xenobiotic response, suggesting that many xenobiotics induce benefits by acting as calorie restriction mimetics [231]. Perhaps of relevance, there is a branch of research that is looking at compounds that appear to have very low toxicity yet appear to induce the adaptive protective response—mainly because they alter membrane structure; it is embraced by the “membrane sensor hypothesis” [232,233]. In this light, it is perhaps relevant that CBD can induce the ER stress response, which can induce apoptosis in hepatic stellate cells [234]. As data are emerging that Nrf2 (and 1) can control the composition of high-density lipoprotein (HDL) [235], which is known, as part of the lipoprotein system to control inflammation [236], it raises the fascinating possibility that compounds like CBD could induce a blood-borne protective anti-inflammatory response. The point here is that the modulation of membranes is tightly linked into the xenobiotic response, but it is also part of the innate homeostatic mechanism.

### 5.3. Membranes and the Electrome: Modulation of Fluidity and Redox

That life is electrical has long been discussed, and although this concept has gone in and out of favour within scientific circles, it is now coming back in favour as new techniques are developed. In fact, it cannot be argued any other way, as life is all about the movement of fundamental charged particles, such as electrons and protons, as well as larger ions, and at the nanometre scale, the fields generated are massive. However, these fields would not exist without membranes, which are also charged. This does suggest that redox-capable lipophilic natural products, such as the phytocannabinoids, are going to alter the “electrome”.

On one level, their lipophilic nature indicates they could modulate multiple “targets”, such as ion channels, or large complex multi-subunit proteins imbedded in membranes. If they evolved to help deal with stress, which disrupts structure that itself relies on controlled dissipation, then it may be that they somehow act to restore dissipation to a homeostatic setpoint. In something as complex as a plant, this may well mean they somehow reset the “morphogenetic” bioelectric field to favour survival. In effect, enhancing dissipation could be an important way to induce formation of a more structured morphogenetic field. Depending on dose, this could mean sacrificing failing cells by enhancing dissipation to a point where the cell kills itself, while inducing adaptation in others but, critically, ensuring cooperation. This same function could also activate immunity, which is essentially a mechanism that relies on telling self from non-self, removing the damaging agents and then encouraging regeneration. However, the same principle could also kill some pathogens; altering their ion channels, and perhaps inducing excessive dissipation, would disrupt their bioelectric fields. At the simplest level, this could be achieved simply by disrupting their membranes, but it could also disrupt the formation of a biofilm by inhibiting cooperative quorum sensing. The same principle can of course be applied to cancer.

But what are they doing at the most fundamental level? What is their real “mode of action”? Potential clues may come from the mechanisms involved in NPQ. Plants must be able to cope with extremes in both heat and light, and when there is too much energy from too many electrons, they have to quench, in effect, dissipate the energy, or risk seriously damaging not just the photosystems but also other membranes and proteins due to oxidative stress. It seems they evolved many ways to do it, ranging from the cycling of compounds like zeaxanthin, use of antioxidant compounds, to maintaining membrane fluidity to allow damaged components to replaced [18]. The latter point is likely key. Membrane fluidity is essential for function, but it is decreased by free radicals due to cross-linking, and if not corrected, makes the membrane stiffer, so not only reducing the ability of proteins to move but also inhibiting signalling. A stiffer membrane can also reduce the flux of molecules like oxygen, potentially worsening the situation—especially in the mitochondrion, as it can potentially lead to more free radicals. In terms of the basic chemistry, membrane stiffness increases with chain length, but is decreased by unsaturation, which also makes it more susceptible to oxidation. Short-chain lipophilic molecules can also increase fluidity. It is thus perhaps relevant that redox-capable lipophilic compounds like ubiquinone are so important in biology [9]. In fact, all living organisms constantly manage lipid fluidity, as their structure is very sensitive to the environment, for instance, temperature or pH. High fluidity enables proper folding of proteins and their movement, but too much can result in increased proton permeability, while too low results in reduced respiration due to hinderance of ubiquinone movement. Certainly in bacteria, low fluidity results in growth arrest and large-scale lipid phase separation into liquid-disordered and gel phased membranes and the loss of many essential functions—especially of the ETC [237]. In short, there is a very tight couple between membrane fluidity, charge flux, redox and thus function. It could be argued that this relationship goes all the way back to the beginnings of life.

Now let us look at CBD. As previously indicated, it is well described to insert within membranes as it is so lipophilic. It also contains a phenol moiety, as well as a methyl group, and so has the ability to quench electrons and transport protons. It thus belongs to a very large class of plant compounds that are viewed as antioxidants. In fact, it is well described to have both direct and indirect antioxidant activity, although how it does this has not always been clear—but it can be anti-inflammatory and can inhibit inflammatory pathways [238]. Critically, both CBD and THC can display their antioxidant capacity independent of cannabinoid receptors and act as neuroprotectants [239]. It is therefore of no surprise that CBD can protect membranes against UVB, which seems to involve both its antioxidant capacity and its ability to modify membranes and alter signalling, with a hint that it could engender enhanced protein turnover [240]. In fact, it is well described that CBD can have both antioxidant and prooxidant activity, where the outcome is dependent on the oxidative stress status of the cell. This seems to be related to its ability to balance the activity of the redox-sensitive factors, Nrf2 and NFkB. In effect, CBD does seem to favourably balance redox to reduce cellular damage but also, where necessary, enhance processes like autophagy, or indeed, induce cell death [224]. It therefore seems to act to restore redox homeostasis. In a way, it can damp down excessive oxidative stress to prevent too much damage but still provide enough stress to induce adaptation.

Overall, this would suggest it acts to manipulate membrane fluidity, both by direct interaction but also by managing redox. The key here is that this effect would be greatest in cells in a state of oxidative stress, where membrane fluidity could be reduced and membrane stress signalling could be permanently switched on. When viewed from the origins perspective, it is likely that this property has become enhanced by the increasing complexity of proteins and signalling systems that through evolution have evolved to fine tune this process and maintain the ability to dissipate energy. As indicated by the NPQ process, membrane fluidity ensures that damaged proteins can be more easily turned over, and if whole organelles are dysfunctional, or even whole cells, cell death can be invoked. Critically, mitochondrial membranes also contain unsaturated fatty acids, in particular, cardiolipin in the inner membrane—which is both highly susceptible to oxidative damage and critical to membrane shape and function. Furthermore, mitochondria tend to contain less saturated than unsaturated fats [241]. This would suggest that compounds like CBD could be critical in mitochondrial protection. But perhaps most importantly, its effects would be biphasic and operate within a Goldilocks zone. As previously indicated, the fine tuning of fluidity could also be achieved by controlling the temperature, which of course ties in well with dissipation.

### 5.4. Cannabinoids “Grease” Membrane Entropy to Restore Homeostasis: Not a Crystal, Not a Fluid—The Self-Correcting “Just Right” Spot 

**Figure 3:**
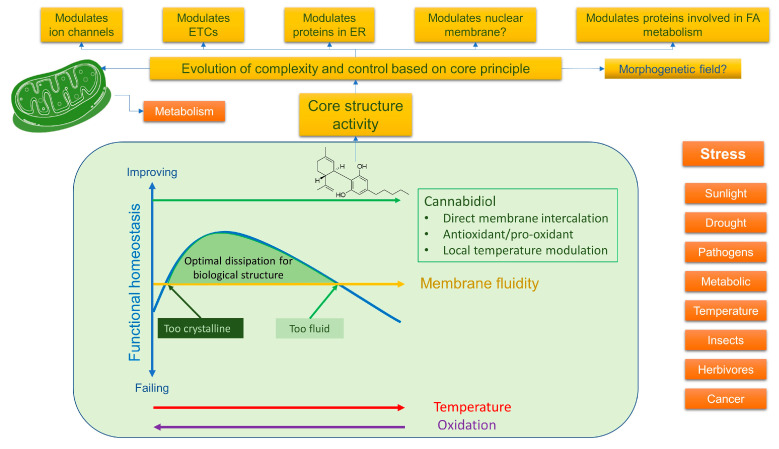
The mode of action of phytocannabinoids, such as CBD, reflects basic physical properties of modulating membrane fluid dynamics and redox resulting in a number of effects, the most basic of which is to control thermodynamic dissipation to maintain functional homeostasis. This is likely to be more nuanced as the plant produces many similar molecules, which would have slightly different effects, perhaps providing better control of membrane fluidity—a kind of “entourage” modulation of membrane dynamics. It could be argued that these are fundamental “prefactor” attributes of these molecules around which the complexity of biology has evolved to enhance homeodynamic control, which explains why it seems to interact with so many “conventional” cellular targets that, say, control calcium signalling. Clearly, the system co-evolved in the plant to help it deal with plant-related stressors, but as plants and animals share fundamental metabolic pathways, and many similar stressors, these compounds can act to restore homeostasis in animals as well. This potentially highlights the existence of a similar stress-signalling membrane fluidity-based homeostatic system in plants and animals—but in animals, we call it the endocannabinoid system. Interestingly, it also suggests, given the role of membranes and their components in controlling cellular cooperation, for instance, via bioelectricity, how they might be working in resolving inflammation and cancer, as well as helping determine the existence of pathogens and providing a means to eliminate them (discussed in Section 6).

As discussed, life is a far from equilibrium self-organising and adaptive structure, exporting disorder to maintain its own identity. It therefore exists at a state between a highly ordered crystal and a totally disordered fluid. One definition of life is that “it is a far from equilibrium self-maintaining chemical system capable of processing, transforming and accumulating information acquired from the environment”, which is grounded in the laws of thermodynamics. A key property inherent in this is the ability to self-correct [242]. One clear example of this is of course the membrane, which is why life works so hard to keep the fluidity in the “sweet spot”. It could be argued that if membranes become too stiff, the system shifts towards a more rigid structure and loses its flexibility to adapt, for instance, by maintaining efficient homeostatic signalling. In effect, it undergoes a phase change, and if this is not altered, it could enter a self-destructive spiral. Indeed, it has been said that from a thermodynamics perspective, ageing is associated with a gradual loss of specific entropy production due to the build-up of irreversible modifications, but this can be modified by occasional stress to induce adaption [243]. But the key observable here is that ageing, in particular of mitochondria, is associated with a decrease in membrane fluidity [244]. More recent models of membrane fluidity changes, involving hydroxyurea, which can induce senescence and invoke reduced membrane fluidity, seem to support a role for this in the development of conditions like Alzheimer’s that are far more likely with increased age [245]. In short, molecules like CBD, especially in combination with others, such as CBG, THC and both their acid and varin forms, may well restore membrane fluidity to the optimum entropic point, so enabling homeostasis (Figure 3). The acid forms could even, perhaps, have a small protonophoric effect. Other compounds, such as beta-caryophyllene, which is also found in the plant, could also be part of this. Overall, the outcome would be biphasic; for example, low doses could enhance fluidity and signalling and, potentially, energy flux, but as doses increase, they would start to inhibit the same processes. So, they would potentially become prooxidant and potentially inhibit energy production. Furthermore, this would also alter cellular cooperation and communication.

This also raises the rather interesting possibility that the endocannabinoid system in animals could also, in some ways, share some of these properties. In short, it could be “endohormetic” [129]. Perhaps significantly, beta-caryophyllene is also a cannabinoid receptor 2 (CB2) agonist and is apparently effective as an anti-inflammatory at a low dose [246]; there are also many “entourage” molecules that also have cannabimimetic properties, including the terpenes, but their targets are not really known [247]. Indeed, it is not just the cannabis plant that contains “cannabinoidergic” compounds, and evidence is also emerging that plants have more conventional lipid signalling moieties to those found in animals [248,249]. Tellingly, “cannabinoids”, as defined by their ability to modulate the CB1 receptor, and this includes THC as well as artificial ones, all have the potential to dissolve in a membrane and alter its structure, but the effect depends on its composition and their structure. For instance, THC can increase fluidity, while AM251, an antagonist, seems to stabilize the bilipid layer [250]. Given that membranes probably evolved long before complex proteins, the existence of lipophilic compounds in both plants and animals that can alter membrane structure is perhaps key in understanding their fundamental actions. That they modulate complex signalling receptors falls naturally out of this.

## 6. Integrating an Optimal Phytocannabinoid Dissipation Model with Disease 

In the preceding sections, we provide a slightly different perspective on how to view disease and why compounds, such as CBD, may work. What has become increasingly clear is that for many compounds, especially natural products, it is not possible to apply the conventional single drug, single target approach as they interact with an ever-expanding list of cellular components. This suggests that we may need to think differently—how biology would have evolved around their core structure—so rather than thinking about the extant concept of a “cofactor”, we need to think of a “prefactor seed complexity” model. Of particular relevance here might be that compounds like CBD are highlighting that there is a tight coupling between ion channels, bioenergetics, stress adaptation and bioelectricity, and the manipulation of membranes.

The reason may well be straight forward; life has evolved from much simpler systems and obeys basic laws, but today, although it appears incredibly complex, the simplicity is still there at its core—so to understand it, we may need to view it in this way. Take, for example, calcium, how is it that one ion can have so many effects? The answer is that it was around before life evolved and is now an essential part of it, as were fundamental particles like electrons and protons. All of these were organised by energy flow and thus the dissipation of energy and with basic chemistry, gave rise to a self-perpetuating and evolving system leading to more complex molecules like proteins and fatty acids, including large polymers that retain information. But what could have been another factor in the organisation? 

As we have mentioned, life is electrical, as it is all about the movement of energy via charged particles, which of course means a tight coupling between the creation of electrical fields through charge separation. And therein perhaps lies the most fundamental interpretation of all—these fields contain information. The movement of these ions creates a field, but the field influences the movement of the ions. The “solid” world of observable molecules is intimately coupled to the invisible world of fields—a molecular yang to a field yin. From this perspective, the existence of a morphogenetic field, where ion channels are pivotal and are linked to bioenergetics, makes this perhaps a much more acceptable notion. Any molecule that can alter membrane structure, and thus ion channels, as well as having a quantum structure that suggests involvement in redox, is going to alter these fields. The classic example of this is the emerging data that quantum “spin”, and thus, free radicals and triplet states, can detect, and be manipulated by, magnetic fields in biology [251].

What is perhaps most telling is that many natural compounds, like CBD, are largely non-toxic, yet can induce a stress adaptation that might enhance survival, indicating that they are manipulating a very fundamental system in life without causing damage. Although it is often said that the dose makes the poison, many natural compounds seem to have a very large therapeutic ratio, which is perhaps thought provoking. In contrast, most man-made drugs, which nature has never seen before, come with a very long list of potential side effects. In this last section, we examine a few examples where compounds like CBD have known efficacy and discuss how a bioenergetic/bioelectric/membrane fluidity viewpoint might explain how it works, for example, in epilepsy, cancer and inflammation, and pathogens, and we finish with a possible universal concept.

### 6.1. Epilepsy

In one respect, epilepsy is the archetypal “electrical” disease. It is one of the most common brain disorders, affecting more than 50 million people worldwide, and has complex and different causes. Unfortunately, anti-epileptic drugs only seem to work in up to 30% of people. During epileptogenesis, neuroinflammation and oxidative stress are self-reinforcing, leading to further brain injury, and are associated not only with changes in the seizure threshold, but also the cognitive and behavioural problems that these patients also experience—including neurodegeneration. These are associated with profound changes in mitochondrial function and ROS production—hinting that measures to control oxidative stress and mitochondria could be valuable [252]. The lack of efficacy of conventional treatments suggests they are not completely modulating the right system. Interestingly, there is growing interest in the link between ion channels and immunity in epilepsy, which is showing some promise [253]. However, there has also been interest in the role of mitochondria in epilepsy for many years [254], which recently was further enhanced because they seem to regulate the neuronal activity setpoint in relation to the calcium buffering activity and thus play a key role in epilepsy [255].

Data suggest that brief stimulation of TRPV1 in sensory neurons leads to a presynaptic elevation of calcium and the release of glutamate, which is dependent on mitochondrial uptake of the calcium [256]. Critically, about 25% of patients with a primary mitochondrial disease display epilepsy, with defective astrocytes playing a key role [257], while prolonged seizure activity impairs mitochondrial bioenergetics leading to cell death [258]. Overall, it appears that mitochondrial function is reduced in epilepsy, leading to a loss in ATP and, in particular, the ability of mitochondria to buffer calcium, and thus predisposing to cell death. This has led to studying transcription factors like Nrf2, which is key in antioxidant defence, and why, for instance, a ketogenic diet is effective, as it enhances mitochondrial function [259]. Nrf2 is key in maintaining mitochondrial health, both in terms of ensuring sufficient antioxidant capacity but also in mitophagy and mitochondrial biogenesis [260].

In fact, it now appears that mitochondrial function could be key in maintaining the neural circuit firing rate setpoint by modulating calcium and energy supply, thus maintaining average activity in the hippocampus. Inhibition of the mitochondrial enzyme, dihydroorotate dehydrogenase (DHDOH), has been shown to reduce spare mitochondrial respiratory capacity but not ATP synthesis. Critically, it enhanced mitochondrial calcium buffering capacity during spiking activity but reduced resting mitochondrial levels; mitochondrial calcium overload has long been thought to be important in epilepsy. In short, it seems that mitochondria play a pivotal role in brain metabolic homeostasis by ensuring the mean firing rate by controlling a wide range of homeostatic effector mechanisms, so making them a therapeutic target in epilepsy—even in difficult to treat conditions such as Dravet’s [261]. Furthermore, it has been shown that genetic reduction in the mitochondrial pyruvate carrier in neurons results in hyperexcitability and propensity to seizures, which was associated with reduced mitochondrial calcium buffering ability and decreased M-type K^+^ channel activity but partially overcome by ketones. Plus, as pyruvate uptake into the TCA cycle is important in the generation of neurotransmitters, reduced oxidative phosphorylation can further result in enhanced hyperexcitability [262]. This certainly supports the central role of mitochondria in calcium signalling [263]. It also of course highlights the close tie between bioenergetics and electric fields.

Overall, it could be argued that this might make the case for optimal mitochondrial function being a key factor in preventing epilepsy. With regards phytocannabinoids, CBD has shown efficacy in both children with Dravet’s [264] and in a mouse model—where it showed clear signs of disease modification [265]. This idea certainly supports the original data and thinking of Ryan et al. [167], who linked CBD’s anti-epileptic effects with mitochondrial modulation of calcium signalling; significantly, they suggested a role for NLCX [167]. When combined with the observations of Rimmerman and colleagues that CBD could be binding VDAC1, resulting in enhanced mitochondrial influx and swelling, which could be alleviated by lowering extracellular calcium [156], and its ability to activate TRPV1 as part of a possible anti-epileptic mechanism [266], it could be argued that a significant proportion of its mode of action involves manipulation of mitochondrial function—both indirectly and then directly. If combined with other data indicating that it can also directly affect the ETC [113,267] and be able to restore mitochondrial function in neurons [211,268], it could both potentially reduce excitability but also induce an adaptive antioxidant response—hence, a degree of disease modification from the inflammatory perspective.

**Figure 4:**
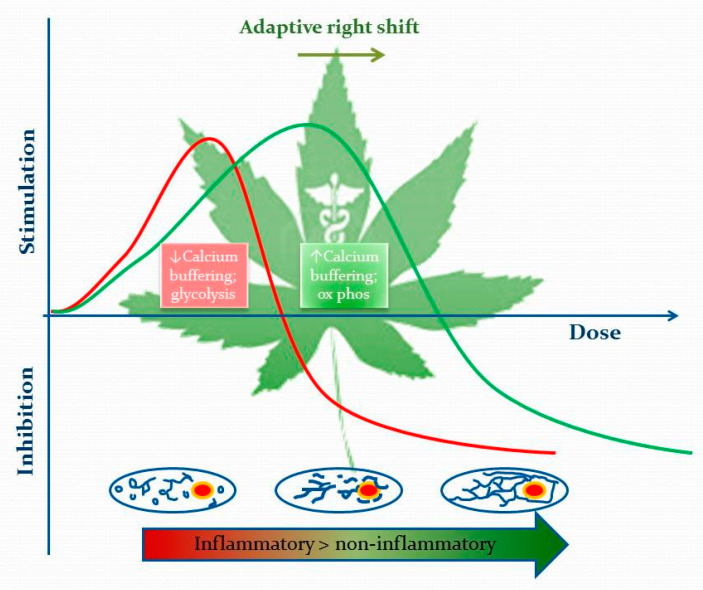
CBD in Dravet’s within the effective dose range. In more conventional terms, it may right shift the cellular biphasic response to stress by upregulation of cytoprotective pathways and enhancing mitochondrial calcium buffering. This moves the system away from an inflammatory vicious cycle towards a more robust phenotype that can deal with an inherent defect of metabolism due to a mutated sodium channel mutation. If, as we believe, cellular bioenergetics and cooperative electric fields are linked, then this could be viewed as a higher-level induction of homeostatic cooperation.

On a practical level, it would appear that CBD could reduce an excitotoxic signal by altering calcium signalling via modulation of the mitochondrion, thus preventing the initiation/continuation of a vicious inflammatory cycle. This cycle will always occur in patients with defective sodium channels, but in the presence of CBD, its progress might be inhibited. Not only would CBD directly initiate a cyto-protective response, but it would also downregulate inflammatory signalling that would ensure normal mitochondrial dynamics become re-established and, thus, normal cellular bioenergetics. Critically, the dose required for this effect would be left shifted in inflamed tissue, making it more sensitive, especially if the ability to buffer calcium was reduced. Restoration of a fully functional mitochondrial system is key in calcium homeostasis. In a sense, by altering calcium homeostasis, not only is there an immediate effect on signalling, but it induces an adaptive response that increases the ability of the cell to control calcium homeostasis. In short, by improving mitochondrial function, the cellular calcium capacitance increases and the biphasic curve is right shifted (Figure 4), bringing the system back into its metabolic flight envelope.

From another viewpoint, it could be restoring a cooperative multicellular electric field. Changing ion channel function can have a profound effect on regeneration [55]; plus, when thinking about how life evolved, for instance, from single-cell prokaryotes to complex-thinking metazoans, the concept of scale-free cognition comes into play with regards to developmental bioelectricity [269]. In effect, it could be argued that epilepsy is what happens when this multicellular cooperation breaks down, but because this happens in the brain, the outcome can be devastating. It seems that a single compound can restore this balance by nullifying the effect of a damaged ion channel. This would suggest a tight coupling between bioenergetics, the morphogenetic field and cellular cooperation. Epilepsy could simply be described as a lack of cooperation between cell types in the brain.

### 6.2. Inflammation and Cancer the (Un)Integrated Flipside of Cooperation

Inflammation can be viewed as an adaptive mechanism to restore homeostasis, but if the system cannot resolve itself, the very same process can result in a vicious cycle and can be viewed from the self-organising dissipative perspective, but at a global scale; a failing system is thus removed. Key in this is that the stress induces a corrective adaptive response, which if it is sufficient, can restore homeostasis and is essentially hormetic [15]. Although evolution has been thought to be mainly about competition, in fact, cooperation is just as important, suggesting that we need to integrate evolutionary and developmental thinking—scale-free biology [270]. Equally, cancer can be viewed as a cooperative problem, where some cells stop cooperating with each other; there could well be genetic components, as well as physiological, but the evidence also points to a key role of a failure of morphogenetic signalling involving bioelectrics [271]. This of course suggests that given the relationship between regeneration, development and cancer, it may well be reprogrammable [27].

Critically, chronic inflammation has long been known as a cause of cancer, as well as promoting it, as regeneration and repair have evolved as more of a priority during evolution, as cancer tends to occur later in life past the reproductive period [272]. There has thus been a lot of interest in treating it using anti-inflammatories, including plant secondary metabolites [273]. In short, many of the processes in cancer and inflammation are similar and thus relate to a failure of regeneration and its control. It could be argued that chronic inflammation and cancer both represent a failure of cellular cooperation. Interestingly, it has been suggested that because ion channels are important in cancer, it could be viewed as an “oncochannelopathy” [274]. In addition, when it comes to dissipation, it could be said that there is a scale-dependent Goldilocks zone for an individual organism, where getting just the right amount of dissipation ensures cellular cooperation—where some cells die for the greater good, while others divide to ensure regeneration of damage. A key component of this “cooperative glue” could well be the bioelectric field due to the information it contains. This is of course dependent on membrane structure.

On a more basic biochemical level, errant calcium signalling is pivotal in inflammation and cancer, and is highlighted by the loss of a tumour suppressor gene, FUS1/TUSC2, which encodes for a mitochondrial protein, leading to reduced mitochondrial calcium accumulation and hyperactivation of factors like NFAT and NF-KB: this is associated with low respiratory reserve, increased inflammation, senescence and accelerated ageing [275]. In effect, the ability of mitochondria to take up, hold and then release calcium at the right rate is key in the suppression of tumours, inflammation and ageing. In fact, it is now becoming clear that rather than mitochondrial outer membrane permeabilization (MOMP) being an all or nothing process, perhaps giving an either/or outcome (for example, non-inflammatory apoptosis versus necrosis/pyroptosis, which are highly inflammatory), it is far more nuanced and carefully controlled, for instance, in what damage-associated molecular pattern (DAMP) entities are released and whether or not caspases initiate cell death or act as anti-inflammatory agents. This nuancing has been honed through evolution due to pathogens [276]. Indeed, it is now becoming accepted that sub-lethal mitochondrial stress can induce a variety of adaptive scenarios by using the apoptotic machinery in a variety of ways [202]. Also of relevance is the role of the NLRP3 inflammasome, which has been shown to be involved in tumour formation in many models; however, there is some evidence that it can also be protective, so it could be somewhat of a double-edged sword [277]. The mitochondrial ETC is now thought to be key in controlling inflammasome activation [278].

With regards to CBD, the fact that it maybe modulating the inflammasome [191] and, more broadly, displays some anti-inflammatory effects [193], as well as having anti-cancer activity, with a variety of mechanisms being suggested [125,126], does continue to indicate that its ability to modulate mitochondrial function is likely to be playing a key role in these effects. Furthermore, and as suggested, if cellular cooperativity and the bioelectricity are interlinked, this might also suggest that compounds like CBD are acting to restore the system to the dissipative Goldilocks zone.

### 6.3. Pathogens

As discussed, compounds like CBD seem to have the ability to induce protection in some cells, while killing others, such as those that have become cancerous. This seems to extend to pathogens. It has been known for many years that both THC and CBD are not only bacteriostatic, but can also exhibit bactericidal activity, with minimum inhibitory concentrations (MIC) at less than 10 µM against some species [141]. Indeed, it seems that many of the phytocannabinoids have anti-bacterial activity, but their chemotype is poorly defined, as is their mechanism of action. However, their lipophilicity and the presence of a phenol group is important, the latter, of course, is well known to have anti-bacterial activity. In effect, the resorcinol moiety is the likely pharmacophore but is modulated by the side chains [140].

Interestingly, CBD has been studied as an add-on therapy with more established antibiotics, such as bacitracin, and has been found to be very effective against Gram-positive bacteria. Of particular interest is that the authors found that CBD could depolarise the cytoplasmatic membrane at extremely low concentrations of less than 0.2 µg/mL [142], which is in the µM range. Further studies support that its mode of action involves modulating bacterial membranes and continue to indicate that CBD seems to also work well against bacterial biofilms and has been shown to be effective against a number of bacterial strains, including those known to cause acne—and is now in phase II trials for this condition. Indeed, data continue to indicate that it has activity against a very broad range of bacteria, including *Neisseria gonorrhoeae*, and seems to have a low capacity to induce resistance. Unfortunately, it seems to have little systemic activity due to its protein binding capacity [279]. Interestingly, in a study looking at using CBD to disrupt *Streptococcus mutans* oral biofilms, the researchers found that it inhibited metabolism but induced hyperpolarisation of the membrane, but still strongly inhibited bacterial growth and biofilm formation [280]. Interestingly, CBDA, the acid form of CBD, seems to be less effective as an anti-microbial agent, probably because it is less permeable in membranes. Other phytocannabinoids, such as cannabigerol (CBG), also have anti-bacterial properties, for instance, against *Stretococcus mutans*, as it decreases membrane fluidity but induces hyperpolarization [281]. CBG also seems to inhibit quorum sensing and biofilm formation in *Vibrio harveyi* [282], suggesting a higher-level anti-pathogen activity that effects cooperation, which as we discuss later, could also be linked to bioelectric effects. This does suggest species-specific effects but also that entourage combination may be important and that both the acid and non-acid forms may have different functions.

Can these compounds also be anti-viral? Clearly, by modulating apoptosis or innate anti-viral mechanisms, or even altering membrane fluidity, they could be, but do they have more direct effects, for instance, by directly binding to viruses? There have been hints; for instance, a study published in 2004 showed that THC could inhibit lytic replication of herpesviruses in vitro [143]. A later study hinted that cannabis use could lower HIV viral load in AIDS sufferers [283]. However, some have questioned the efficacy, as there were very little reliable data up to 2020 [284].

This changed somewhat with the emergence of SARS-CoV-2. Fairly soon after the pandemic started, one group hypothesised the potential benefits of CBD, as it could potentially inhibit the cytokine storm and lung inflammation, possibly via agonism at PPARγ, and in particular, as there was some evidence it could downregulate angiotensin-converting enzyme 2 (ACE-2) and transmembrane serine protease 2 (TMPRSS2) expression [285]. We also suggested that CBD could be efficacious, as it is likely that the virus modulates mitochondrial function [286]. It was then found that CBD and THC could bind to SARS-CoV-2 M^pro^, its main protease, which is thought to have a key role in viral replication. In combination with CBD’s known anti-inflammatory actions, it was again suggested as a possible treatment [287]. Further studies identified that the acid forms of CBD, CBDA and cannabigerolic acid (CBGA), were also able to bind to the virus’s spike protein and inhibit its entry into mammalian cells [288].

The overall consensus is that the plant secondary metabolites, such as the cannabinoids, evolved to help the plant resist stress. By definition, they must enhance robustness across multiple systems to many different stressors, ranging from excess light, especially UV, to drought, herbivores, insects and, in particular, pathogens. At its core, this resistance must involve bioenergetics, as energy is key to life by way of dissipation, which in turn is about information—but misdirected energy, for instance, as oxidative stress, can seriously damage an organism. Pathogens can represent a serious threat as they can disrupt this balanced system. This might suggest that the same mechanism used by the host organism to survive stress by managing dissipation could also be used limit pathogen replication, which, after all, is another form of dissipation. For example, excessive dissipation of the ion gradient across a bacterial membrane will rapidly kill it or at least break up a biofilm; equally, inducing excessive dissipation will kill its host cell but limit the infection; and an intermediate level of dissipation may inhibit viral uptake. Maintaining the right level of dissipation in a multicellular component could be key in recognising self from non-self and, in particular, providing information about regeneration following infection. As we have suggested, if one of the most fundamental properties of secondary plant metabolites, and of their precursors before eukaryotes evolved, was to act as sunscreens, which work by dissipating excess energy, then one could argue that this fundamental principle was built upon as complex life evolved [10]. A possible example of this is that plants use uncoupling proteins to protect against stress, coupling bioenergetics, signalling and the stress response; although they can be involved in thermogenesis, they also control redox and metabolite flow—in effect, they “flexibilize” cellular metabolism [89].

There is perhaps also another way of looking at this: recognising self from non-self and the importance of controlling cellular cooperation and the role of bioelectric fields, which are, of course, intimately coupled to membrane function. Compounds like CBD could simply help an organism, or a sub-organismal cellular cooperative, such as an organ, maintain its identity and thus recognise the threat from invading pathogens. The similarities to how it might also deal with cancer here are clear.

## 7. Conclusions

We may need to view how compounds like CBD work through the lens of evolution and the basic laws that govern our universe.

In this paper, we have gone back in time and reflected on the fact that life must have evolved from something much simpler but followed the basic laws of physics. Thus, although it seems very complex now, it was not so in the past, and it started because the chemicals available on the early Earth were organised by an energy gradient into a dissipating structure, which through natural selection became more complex and stable. The fact that it relies on many iron/copper-based structures, as well as aromatics, and the formation of ion gradients across membranes is key. This is perhaps the most powerful application of Occam’s razor. Equally, it is also important to embrace less established but very logical theories, such as hormesis and the importance of electric fields in biology, which naturally fall out of adaptive thermodynamics and quantum mechanics. At the same time, we need to reposition the relative importance of the disciplines of “established” fields like pharmacology and genetics. As ever, how a researcher views and weights the importance of a particular subject is heavily influenced by the zeitgeist of their time.

Today, we need to move towards a polymath integrated approach. For example, it is well described that most, if not all, cellular systems are integrated, for instance, there is a close association between the cytoskeleton, ion channels and mitochondria in controlling neuronal excitability [289]. This is entirely logical. Evidence is also that both plants and animals utilise a bioelectric field, which of course requires energy. This would suggest that mitochondrial function, ion channels, membranes, the cytoskeleton and the presence of a bioelectric field are integrated. If we accept that life is a dissipating far from equilibrium self-organising structure, in effect, an island of negentropy, then something that can restore a system to a safe setpoint when the system is under stress could be viewed as a medicine. Hence the continuing need to embrace adaptive thermodynamics and quantum mechanics. By definition, a good medicine would have to modulate multiple systems at once, which would suggest it is acting at a very fundamental level. One such target is of course the charged membrane, and it could be argued, because of the high concentration of membrane bound complexes involved in electron transport, that this certainly involves the mitochondrion. As Nick Lane has suggested, the mitochondrion needs to be viewed as a “flux capacitor” [7].

So, could we ascribe some “fundamental” level approach to a compound like CBD? One of the simplest mechanisms that could be suggested is that by altering membrane structure and/or protein interaction with lipids and redox, these compounds modulate dissipation by altering the flow of charge. If, as is becoming apparent, biology is reliant on quantum principles to function, then any general alterations in membrane structure will have a biological effect, for instance, in altering ion or electron flow through tunnelling, where tiny alterations in distance will have a profound effect. The interesting thing is that compounds like CBD seem to induce, overall, a more stabilised system where redox is better controlled—this could only be explained by an integrated rerouting of energy to systems evolved to quench it safely, coupled with an upregulation of these systems to ensure future robustness, so ensuring homeostasis. Central to this would be membrane fluidity. Critically, however, this only operates within a Goldilocks zone; if the system is too damaged or the dose is too high, this rerouting becomes lethal, hence there are tipping points and thresholds.

What is interesting here is that multicellularity does enable an organism to be more robust, as natural selection of subcomponents, cooperation and cell death, as well as specialisation, ensure survival of the whole. So, it could be argued that in general, compounds like CBD evolved to benefit their host but not individual organisms, such as pathogens, although even here, given the importance of microbiota, there could be differential effects. Prokaryotes are far more sociable and usually exist in colonies, such as biofilms. So, disrupting these, in effect, reducing their cooperativity, is a mode of action—although potentially also inducing cooperation in others. It could also be argued that the prokaryotic descendent in our cells, the mitochondrion, is also affected in the same way—perhaps by stress testing them, inducing their death in some cells but enhancing robustness in others. Evidence is that mitochondria do communicate with their distant microbiota cousins, for instance, during exercise [290].

This also leads to the possibility that compounds like CBD are also manipulating the bioelectric field, which helps to engender cooperation in the face of a challenge. Divided we fall, united we survive. Hence, it could be argued that their molecular ability to dissipate, through evolution, has become incorporated and amplified in multicellular organisms to restore homeostasis. Their lipophilicity and phenol group perhaps indicate how they do it. From this viewpoint, it could explain how they work in cancer, inflammation, epilepsy and as anti-pathogens. They may also be telling us what some of these conditions, such as cancer, actually are. If cancer is a reversion to a non-cooperative, every cell for itself state, re-establishing cooperativity, or reactivating the built in self-destruct system for wayward cells, is clearly a prerogative.

Because of their effects on pathogens, scientists have looked at their effects on microbiota. For instance, there is evidence that a mix of THC and CBD, by altering the gut microbiome, can have beneficial effects in a model of autoimmune encephalomyelitis [291], while THC, via a mechanism that may involve modifying both the lung and gut microbiota, can protect against acute respiratory distress syndrome [292]. However, in a mouse model of non-alcoholic fatty liver disease, researchers found that THC-rich extracts benefited, but CBD-rich extracts worsened the condition, both of which were associated with changes in the microbiota [293].

There is, however, still a partly unanswered question: what do they do in the plant, and what is their primary role? Compounds like THCA and CBDA are primarily found in the trichomes and certainly at higher concentrations have anti-pathogen and likely anti-insect activity [1]. Indeed, many of the compounds in the plant do have anti-viral activity [294]. They could even act as sunscreens [295]; THC, CBD and CBN all absorb at about 280 nm and exhibit fluorescence in the 330 nm range—although the former changes a bit after irradiation, the latter changes a lot towards 360–400 nm—suggesting the generation of new species [296]. Whether or not this is their primary role is perhaps still unclear, but they are certainly dissipating energy. Indeed, it is possible that they do not need to directly absorb UV light to protect membranes but can, by buffering excess redox swings, still provide protection—as has been shown in UVB-irradiated keratinocytes [240]. Away from sunlight, inside a cell, redox modulation by chemiexcitation during metabolism is well described [17], so the same principle could be applied.

It could also be argued that if they fall onto leaves, they could induce cell death, but at lower concentrations, if they were absorbed, they could certainly enhance robustness and affect signalling—for instance, by interacting with ion channels and mitochondria and potentially altering the morphogenetic field and/or generalised bioelectrical signalling. They could also alter the plant’s own microbiome. THC, because of its effects on innate animal receptors, could certainly play a role, although what is not quite clear is whether it would make herbivores eat the plants. But if these compounds got into the ground around the plants, they would also alter the soil microbiome and perhaps fungal networks, with interesting consequences; they enhance some but kill off others.

Overall, although there is some conjecture about the primary role of cannabinoids in the plant, their overall function would fit with resistance against several forms of stress, as is generally accepted for the role of secondary plant metabolites. This would suggest that their medicinal properties would be based on exactly the same mechanism. Although many still favour the idea that they work through the endocannabinoid system, it is only likely to be part of the story. But perhaps by embracing some very fundamental principles about the origins of life, thermodynamics and quantum mechanics, and other less explored fields that can be used to explain how they work, we may be a bit closer—especially if we release ourselves from the shackles of established pharmacological thinking. Life likely started, and is based on, the dissipation of energy via the movement of charge through a membrane, which defines its structure and means that electric fields are part and parcel of homeostasis. It would be extraordinary if compounds like the phytocannabinoids, which are based on lipophilic phenolic moieties, are not acting to restore homeostasis via manipulation of this system.
